# Revolutionizing Cancer Immunotherapy: Emerging Nanotechnology-Driven Drug Delivery Systems for Enhanced Therapeutic Efficacy

**DOI:** 10.1021/acsmeasuresciau.4c00062

**Published:** 2024-11-15

**Authors:** Panneerselvam Theivendren, Selvaraj Kunjiappan, Parasuraman Pavadai, Kaveena Ravi, Anusuya Murugavel, Avinash Dayalan, A. Santhana Krishna Kumar

**Affiliations:** † Department of Pharmaceutical Chemistry, Swamy Vivekanandha College of Pharmacy, Elayampalayam 637205, Namakkal, Tamil Nadu, India; ‡ Department of Biotechnology, Kalasalingam Academy of Research and Education, Krishnankoil 626126, Tamil Nadu, India; § Department of Pharmaceutical Chemistry, Faculty of Pharmacy, M.S. Ramaiah University of Applied Sciences, M. S. R. Nagar, Bengaluru 560054, Karnataka, India; ∥ Department of Pharmaceutics, Swamy Vivekananda College of Pharmacy, Elayampalayam 637205, Namakkal, Tamil Nadu, India; ⊥ Center for Global Health Research, Saveetha Medical College, Saveetha Institute of Medical and Technical Sciences, Chennai 602105, Tamil Nadu, India; # Department of Chemistry, National Sun Yat-sen University, No. 70, Lien-hai Road, Gushan District, Kaohsiung City 80424, Taiwan; 7 Department of Chemistry, Saveetha School of Engineering, Saveetha Institute of Medical and Technical Sciences (SIMATS), Saveetha University, Chennai 602105, Tamil Nadu, India

**Keywords:** Cancer, Immunotherapy, Tumor microenvironment, Cancer vaccines Drug delivery, Nanodrug delivery, Target specificity, Clinical trials, Reduced side effects

## Abstract

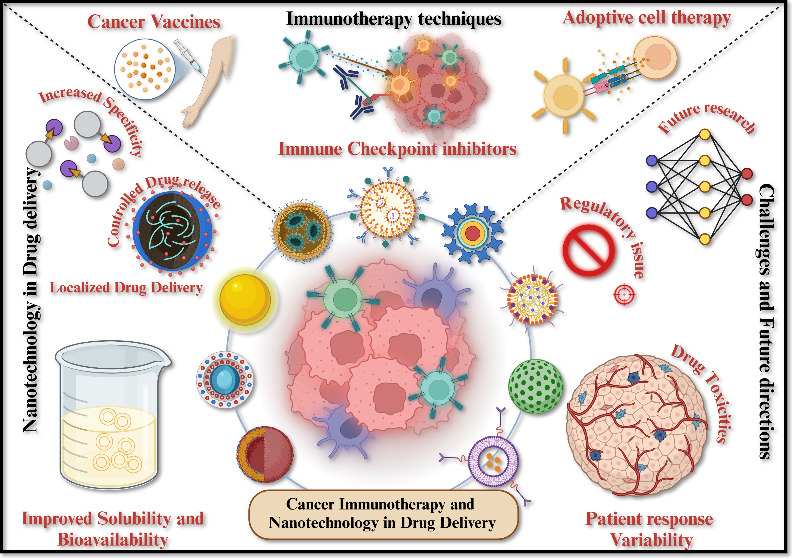

Cancer immunotherapy is an innovative way of treating cancer by stimulating individual cells to overcome cancer. Widespread biomedical studies were carried out with the aim of exploring immunotherapy cancer therapeutics, and this review spotlights some mechanisms in which it was developed, namely immune checkpoint inhibitors (E.G PD-1/PD-L1, CTLA-4), adoptive cell therapy (e.g., CAR T-cell therapy), and cancer vaccines. Although it has shown clinical benefit in a number of cancer types, including melanoma and non-small-cell lung cancer, several challenges have dampened enthusiasm for this approach, from the differing patient response rates to toxicities. Nanotechnology in drug delivery systems must play a role in overcoming the same. Nanotechnology enables increased specificity and controlled drug release, improved solubility and bioavailability, can treat the tumor specifically, and localized drug delivery at the disease site decreases systemic toxicity. The review also features advances in the construction of lipid-based, polymeric, and inorganic nanoparticles that improve drug stability and allow the delivery of cotherapeutic agents. Nanotechnology-based delivery systems can be used alone or in combination with immunotherapy to assist in improving the immune response, gaining access to the tumor microenvironment, and overcoming biological barriers. Thus, the nano-DDS were both safe and effective in preclinical studies, and ongoing clinical trials have shown that they are capable of increasing the therapeutic index of anticancer drugs. Lastly, the review also discusses current challenges and regulatory issues in advancing these technologies and highlights the importance of further research to devise appropriate methodology for efficient functionalization of nanotechnology for individualized cancer solutions in cancer treatment.

## 1 Introduction

### 1.1 Overview of Cancer Immunotherapy

Cancer immunotherapy is a novel approach to cancer treatment that harnesses the body’s immune system to more effectively recognize and combat cancerous cells.^1,2^ Traditional cancer treatments, including surgery, chemotherapy, and radiation, primarily target cancer cells by directly destroying them. Nevertheless, they can also affect healthy cells, leading to significant undesirable responses. Immunotherapy, in contrast, seeks to harness and augment the body’s own immune system, offering a more targeted and perhaps less harmful method of treatment. The main objective of cancer immunotherapy is to enhance the immune system’s ability to recognize and eliminate cancer cells.^3,4^ With its several components-T cells, B cells, and natural killer cells-the immune system plays a crucial role in identifying and destroying aberrant cells. Still, cancer cells often find ways to hide immune detection or stifle immunological responses. Immunotherapy aims to solve these issues by means of several strategies to boost or restore the capacity of the immune system to fight cancer.^5^ PD-1, PD-L1, and CTLA-4 are some of the proteins that these drugs mess with. These proteins normally keep immune reactions in check. T cells fight cancer cells more effectively with checkpoint inhibitors because they stop these proteins from working. Checkpoint inhibitors are drugs like Pembrolizumab (Keytruda) and Nivolumab (Opdivo) that are well-known. They have worked really well to treat melanoma, non-small-cell lung cancer, and bladder cancer, among others. To treat cancer, adoptive cell therapy is a very important method. Scientists take immunity cells from a patient, change or improve them in a lab, and then put them back into the patient to help their body fight cancer cells better. One important change to this method is chimeric antigen receptor (CAR) T-cell treatment. CAR T-cell treatment changes T cells’ genes so they have receptors that can specifically attach to antigens on cancer cells. This kills the cancer cells that have those antigens. Some types of blood cancer, like acute lymphoblastic leukemia and non-Hodgkin lymphoma, have responded very well to this treatment.^6^ The use of cancer medicines is a new way to treat immune problems. These vaccines work by stimulating the immune system to find and attack antigens that are special to cancer. There are two main types of cancer vaccines: those that prevent cancer and those that treat cancer. Vaccines that protect against cancer, like the human papillomavirus (HPV) vaccine for cervical cancer, are called preventative vaccines.

**Figure 1:**
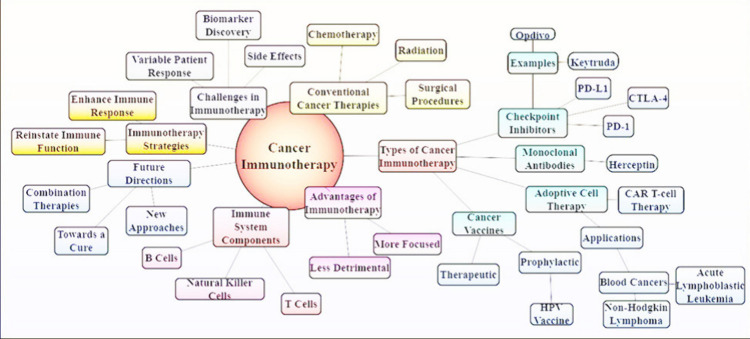
Cancer immunotherapy utilizes the immune system to specifically target cancer cells, employing techniques such as checkpoint inhibitors, CAR T-cell therapy, and vaccinations. This approach provides a less hazardous alternative to conventional treatments.

**Figure 2:**
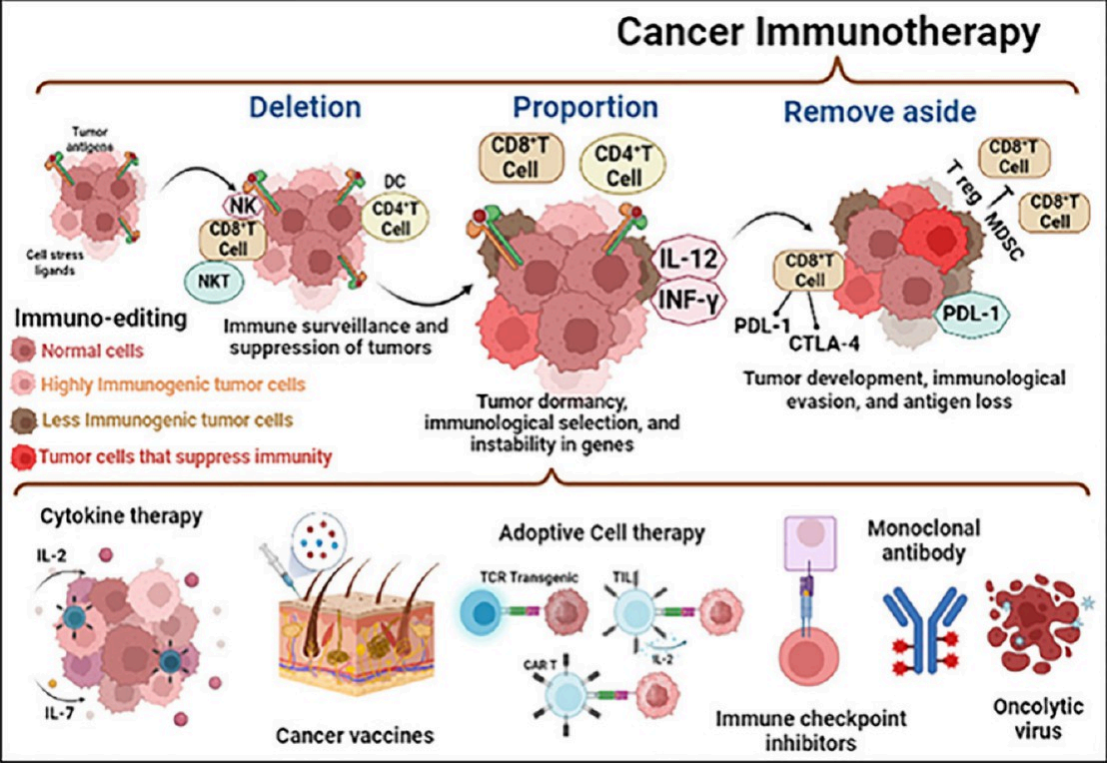
Illustration depicts the several approaches to cancer immunotherapy, such as immune system modulators, CAR-T cell treatment, immune checkpoint inhibitors, and cancer vaccines. These methods improve patient outcomes for a variety of cancer varieties by strengthening the body’s immune response to identify and fight cancer cells.

Conversely, therapeutic vaccines are designed to improve the immune system’s capacity to combat cancer cells in order to treat pre-existing malignancies. In the field of cancer immunotherapy, monoclonal antibodies are indispensable.^7^ These artificial chemicals possess the capability to bind to specific proteins present on cancer cells or other molecules inside the tumor microenvironment. This attachment either marks the cancer cells for removal or inhibits signals that promote tumor growth. Trastuzumab (Herceptin) selectively targets the HER2 protein expressed on specific breast cancer cells, leading to improved outcomes for persons diagnosed with HER2-positive breast cancer. Despite notable progress in cancer immunotherapy, it continues to encounter specific challenges. Not all patients exhibit a favorable response to these medications, and specific individuals may experience adverse side effects. Scientists are presently involved in endeavors to identify biomarkers that can precisely forecast the response to immunotherapy and to develop techniques to enhance the effectiveness and safety of this treatment.^8^ Cancer immunotherapy is an innovative approach in the field of oncology that offers hope for enhanced and personalized cancer treatment Figure 1 and 2. Advancing research in the field of immunotherapy holds the promise of significantly improving outcomes for cancer patients and bringing us closer to discovering a cure. This can be achieved by integrating immunotherapy with current treatment modalities and developing novel ways.^9,10^

#### 1.1.1 Importance of Cancer Immunotherapy

Cancer immunotherapy is a therapeutic strategy that utilizes the body’s immune system to recognize and eradicate cancer cells. Immunotherapy distinguishes itself from conventional medications by specifically enhancing or restoring the immune system’s intrinsic ability to identify and combat malignancies, rather than directly attacking cancer cells. This approach includes a variety of approaches. Checkpoint inhibitors, for instance, impede the activity of proteins that dampen immune responses. Adoptive cell therapy enhances the ability of immune cells to specifically attack cancer cells. Additionally, cancer vaccines exist. They enhance the immune system’s response to target and eliminate cancer-specific antigens. The importance of cancer immunotherapy lies in its ability to offer more targeted and less detrimental treatment options compared to traditional methods. Immunotherapy can yield significant therapeutic results, particularly in cancers that do not respond to conventional treatments, by specifically addressing the immune system’s role in fighting cancer. Moreover, it fosters prospects for personalized healthcare, allowing treatments to be tailored to individual patients based on their unique immune profiles and tumor characteristics. Advancements in research have revealed the promise of cancer immunotherapy to improve survival rates and reduce the occurrence of side effects. In the end, it offers a more effective approach to cancer treatment.^3,10,11^

#### 1.1.2 Current Challenges and Limitations in Cancer Treatment

The current landscape of cancer treatment faces numerous challenges and limits. Though medicines have come a long way, the efficiency of treatments is often hampered by the diversity of malignancies. These can vary greatly between individuals and even within the same tumor. Such variation makes it hard to create uniformly effective treatments. Another major issue is the evolution of cancer cells and their resistance to conventional treatments. This can result in illness recurrence or progression. Although chemotherapy and radiation are well-established treatments, they can cause serious side effects that reduce patients’ quality of life. Thus, there is a need to achieve a balance between efficacy and patient acceptability. An obstacle is the limited capacity to specifically target cancer cells (which harms normal tissues), resulting in systemic toxicity and further difficulties. Furthermore, the high prices of innovative medicines such as targeted therapies and immunotherapies serve as hurdles, making them less accessible to many patients. We also need improved markers for predicting treatment outcomes and tailoring drugs to particular patients. The complexity of the cancer genome and tumor microenvironment also make it difficult to develop effective treatments. Thus, continued research and innovation in this field are critical.^3,4,12^

#### 1.1.3 Therapeutic Effects of Nanoparticles in Cancer Immunotherapy

Now-up-a-days used in cancer immunotherapy, NPs are found to prolong the therapeutic efficacy of drugs via targeted delivery and improved bioavailability. They can help to package therapeutic agents, such as chemotherapeutic drugs, immune checkpoint inhibitors and cancer vaccines and keep these agents stable and release them accurately in tumor sites. The targeted delivery reduces systemic toxicity and off-target effects, thus reducing the side effects of cancer treatment.

The capability to surmount biological barriers, such as the tumor microenvironment that traditional therapies cannot penetrate, is one of the predominant therapeutic benefits of nanoparticles.^13,14^ Nanoparticulate formulations of drugs can be purposefully designed to increase drug accumulation in tumors and take advantage of the enhanced permeability and retention (EPR) effect, localizing delivery of the drug into cancer tissues so as to increase therapeutic responses. Nanoparticles can also be designed to sense the environment with in the tumor such as changes in pH or temperature and release drugs only at that site.^15^

In addition, nanoparticles allow simultaneous delivery of multiple drugs (e.g., immunomodulators and chemotherapy) in a single platform. Together, these strategies work together to enhance the killing power of the immune system against cancer cells and circumvent treatment resistance, which will ultimately translate into improved personalized cancer therapies.^16^

### 1.2 Role of Drug Delivery Systems (DDS)

**Figure 3:**
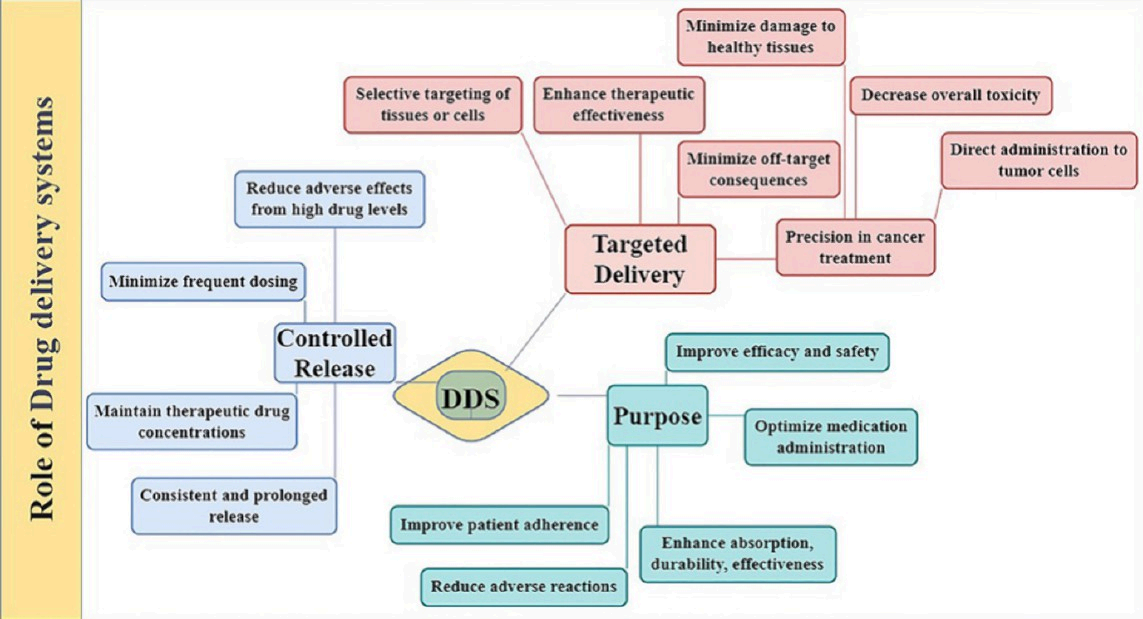
Drug delivery systems (DDS) are designed to optimize the administration of drugs by assuring regulated and sustained release, in addition to focused distribution and a reduction in unwanted effects.

**Figure 4:**
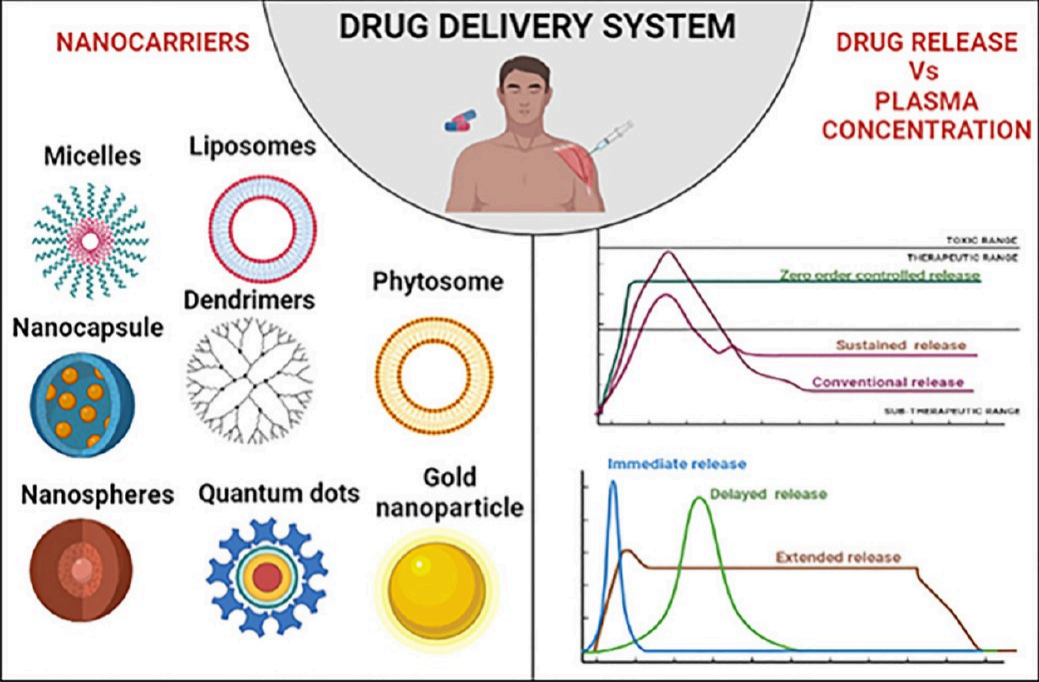
Picture illustrates how sophisticated nanocarriers are being developed to improve medicines’ targeted distribution and controlled release. More effective and individualized cancer therapies are now possible because to innovations in the areas of precise drug release mechanisms, enhanced targeting of cancer cells, and integration with immunotherapeutic agents.

Drug delivery systems (DDS) are very important for making medicinal treatments more effective and safer by delivering medicines to specific parts of the body. These systems are specifically made to improve the absorption, longevity, and usefulness of medicines while also lowering side effects and getting patients to take their medicines as prescribed. DDS is a key part of making sure that medicines are released slowly and safely. Traditional ways of giving medicine often require frequent doses, which can cause drug levels to change and adverse responses to get worse.^17^ Conversely, DDS can provide a steady and continuous release of drugs, therefore preserving therapeutic drug concentrations in the body and so reducing the need for regular administration. This controlled release not only increases the efficacy of the medicine but also lowers the possibility of side effects connected to excessive drug levels in the body. DDS have a clear benefit in that they can specifically target some tissues or cells. Targeted DDS are designed especially to get drugs straight to the desired location of action, hence improving therapeutic efficacy and reducing off-target effects.^18,19^ Precision is critical in the treatment of diseases such as cancer because it allows for the direct distribution of chemotherapy drugs to tumor cells, while limiting damage to healthy tissues and decreasing overall toxicity Figure 3 and 4. Many approaches are used for precision targeting, such as ligand–receptor interactions, antibody–drug conjugates, and nanoparticle-based carriers. DDS also addresses difficulties such as pharmaceutical stability and solubility. A number of drugs show low solubility in water-based conditions, which can impair their absorption and efficacy.^20^ DDS can make medicines more stable and easier to dissolve in a number of ways, such as by containing them in nanoparticles, liposomes, or other carriers. These processes keep the medicine from going bad, make it easier for the body to dissolve, and help the body use it. DDS can also help patients stick to their treatment plans and make things easier for them. Conventional oral drugs often need to be taken more than once a day, which can be hard for patients and lead to not following the directions. Drug delivery methods like transdermal patches, injectable microspheres, or implanted devices can lower the number of times a dose needs to be taken and improve adherence. Medications can be delivered continuously and continuously through the skin with transdermal patches like the one that was described. This lets people wait longer between doses and makes things easier for them.^21^ Modern DDS increasingly incorporate advanced technology such as stimuli-responsive devices. These systems dispense pharmaceuticals in response to specific environmental stimuli, such as variations in pH, temperature fluctuations, or the detection of particular biomarkers. These techniques enable enhanced accuracy in regulating the administration of medications, hence enhancing therapeutic outcomes and reducing adverse effects. Despite the advantages offered by DDS, their application faces numerous hurdles. The complexity associated with the design and manufacturing of DDS might lead to higher costs, which may limit their availability.^19,22^ There is also a chance that the medicine and the way it is given could combine in ways that are not expected, so a lot of testing and confirmation is needed to make sure that everything is safe and works well. Regulatory hurdles and the need for thorough clinical trials could slow down the creation and approval of new DDS technologies. To sum up, DDS are a big step forward in medicine. They offer many benefits, such as controlled release, accurate administration, longer longevity, and better patient compliance. As science and technology improve, DDS may be able to get around the problems that currently present in drug therapy. This could lead to better and more personalized treatments for many diseases.

#### 1.2.1 Importance of Effective Drug Delivery in Immunotherapy

**Figure 5:**
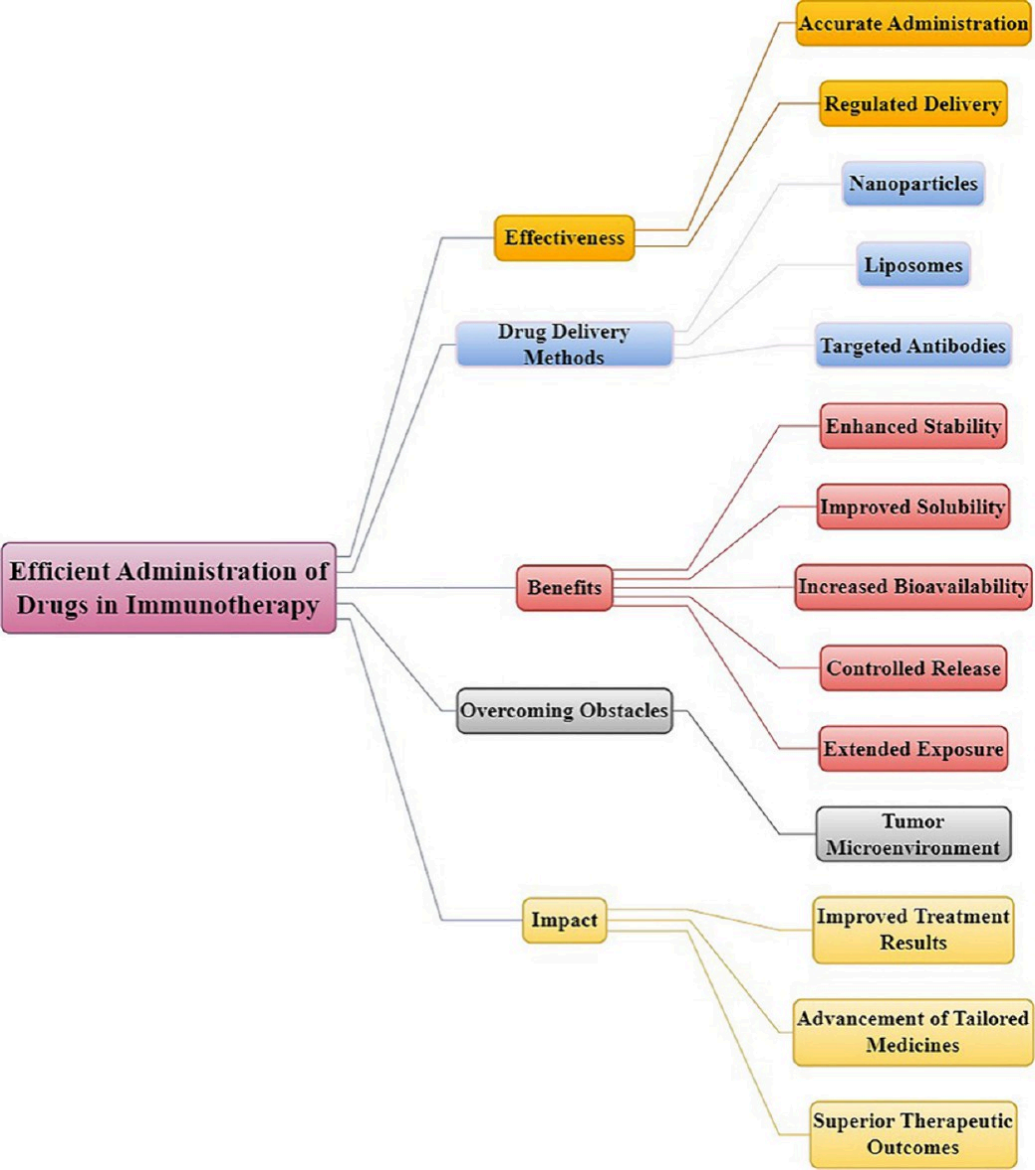
Optimizing drug delivery in immunotherapy involves enhancing drug stability, solubility, and bioavailability through the utilization of techniques such as nanoparticles and liposomes. These methods aim to improve drug targeting, minimize toxicity, and overcome biological barriers, ultimately leading to more effective treatment results.

In immunotherapy, a treatment method based on the body’s immune system to precisely destroy cancer cells, effective drug delivery is absolutely vital. While reducing unexpected outcomes and enhancing efficacy, the effectiveness of immunotherapy is much dependent on the accurate and controlled delivery of therapeutic medicines to the designated sites. Monoclonal antibodies, checkpoint inhibitors, and cytokines are among the immunotherapeutic medicines whose stability, solubility, and bioavailability might be improved by effective drug delivery systems. Modern delivery techniques including nanoparticles, liposomes, and tailored antibodies help researchers to acquire more exact targeting of cancer cells and reduce general toxicity. Furthermore, these methods can allow the drug to be released under control and for a longer period, thereby increasing the exposure to the chosen target and maybe improving the therapeutic response generally. Furthermore, effective drug delivery can help to overcome biological challenges such the tumor microenvironment, which usually prevents the penetration and effectiveness of therapeutic agents. Figure 5. In immunotherapy, optimizing drug delivery benefits both treatment outcomes and helps to promote customized and potent cancer treatments. Targeting and improving drug distribution will help to advance immunotherapy and give patients better therapeutic results.^23^

#### 1.2.2 Limitations of Traditional Drug Delivery Methods

Even though conventional medication delivery methods are an important part of modern medicine, they have some problems that make them less effective and worse for patients. One big problem is that they are not good at controlling the release of medications or being clear about what they do. Traditional ways usually offer a steady discharge rate that might not match the body’s changing needs, which can lead to worse therapeutic outcomes or more harmful effects. Also, these methods often have problems with low bioavailability, which means that medicines do not work as well because they are not absorbed well or are broken down too quickly. This problem is especially important for medicines that need to be taken exactly as prescribed for them to work best.^12^

One other restriction is the invasive character of some traditional methods. Oral and injectable dosage forms could cause patient discomfort, annoyance, or noncompliance, therefore affecting their adherence to treatment plans. Traditional delivery methods do not always do a good job of getting drugs to the right tissues or cells. This can make treatments less effective and raise the risk of systemic side effects. There’s also another matter. Some drugs may not work as well or as dangerously when they are made in traditional ways because they are volatile. These problems show how important it is to have complicated drug delivery methods that make things more accurate and under control. They also want to improve patients’ loyalty and reduce side effects by making these things better.^19,24^

### 1.3 Introduction to Nanotechnology in Drug Delivery

Nanotechnology is an innovative field that dramatically changes how medicines are delivered and controlled inside the body. It uses ideas from physics, chemistry, biology, and engineering to control matter at the nanoscale, which is usually between 1 and 100 nm.^19,25^ Moving things around on such a microscopic level could make a lot of different activities better. Nanotechnology opens up a lot of possibilities for drug delivery because it gets around problems with older methods, like the fact that medicines do not dissolve well or spread out evenly.

One big benefit of nanotechnology for drug distribution is that it can make medicines that do not dissolve well better at doing their job and making them more bioavailable. A lot of pharmaceutical chemicals do not dissolve well in water, which makes them hard to absorb and less effective as medicine. Nanoparticles can make the surface area of these drugs bigger, which speeds up the rate at which they break down and makes uptake better. Nanoprecipitation and liquid evaporation are methods used to create nanoparticles of exact size and shape, which makes the drug more stable and easier to dissolve.^19,25^ By creating nanoparticles that can specifically find and attach to certain cells or tissues, drug delivery systems can effectively lower toxicity and side effects that were not intended. The targeted approach is especially helpful for treating diseases like cancer because nanoparticles can be made to connect specifically to cancer cells. This way, harmful drugs can be delivered directly to the tumor site while healthy tissues are kept safe.^19,25^

**Figure 6:**
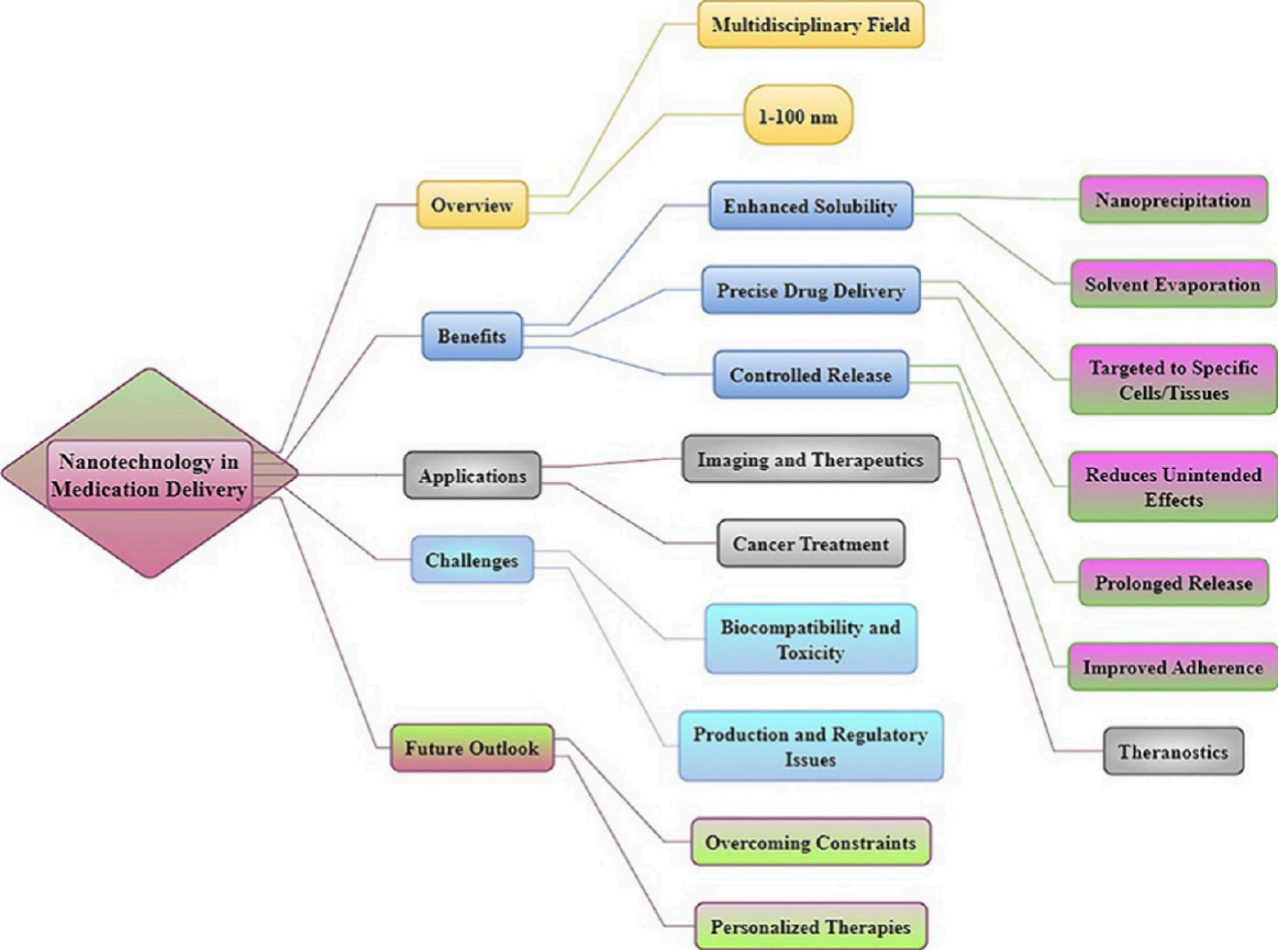
Nanotechnology revolutionizes the administration of medication by improving solubility, allowing for accurate targeting, and permitting controlled release. The system combines diagnostic and therapeutic capabilities, but encounters difficulties in ensuring safety and obtaining regulatory approval.

Furthermore, nanotechnology helps medications be released under control and for a longer duration, therefore enhancing patient adherence and therapeutic effects. By means of controlled release of their contents over extended periods of time, nanoparticles can be designed to minimize the need for regular administration and guarantee consistent therapeutic drug concentrations within the body. This approach lowers the bad effects connected to different drug concentrations and increases the efficacy of the treatment. Extremely flexible, nanotechnology allows one to produce nanoparticles with multiple uses, including imaging and medicinal ones. By fitting imaging agents on these nanoparticles, drug distribution and therapeutic response can be tracked real-time. Theranostics is the mix of therapeutic and diagnostic powers that enhances treatment accuracy and provides vital data regarding the success of the therapy. While nanotechnology offers significant possibilities for the delivery of medicine, certain issues must be addressed as well. Together with the nuances of their manufacture and regulatory licensing, the comprehensive evaluation of problems concerning the biocompatibility and toxicity of nanoparticles is essential Figure 6. Comprehensive research and strict testing help to ensure the safety and efficacy of nanomedicines by means of appropriate standards and norms development. In essence, the application of nanotechnology in medicine delivery presents a novel approach capable of overcoming many limitations connected with traditional drug delivery systems. Although the future development and application of nanotechnology will depend on overcoming the challenges related to safety, manufacturing, and governance, it helps to improve solubility, enables exact and controlled release, and integrates diagnostic and therapeutic capabilities, so opening possibilities for more efficient and customized therapies.^2,26^

#### 1.3.1 Definition and Principles of Nanotechnology in Medicine

Nanotechnology Using nanotechnology in medicine involves employing materials and tools at the nanoscale to detect, treat, and prevent illnesses. This technique leverages the unique characteristics of materials at this tiny scale (1–100 nm), achieving advances not possible with larger materials. Such properties include greater responsiveness, larger surface areas, and quantum events

*Precise Delivery*: Nanoparticles can transport medicines directly to specific cells or tissues, minimizing side effects and boosting treatment efficacy. Modifying the particle surfaces aids in binding to disease-specific markers, enabling targeted delivery. *Enhanced Diagnostic Tools*: It allows for highly sensitive diagnostics, like nanosensors and imaging agents, capable of detecting diseases earlier and more accurately. *Personalized Medicine*: Tailoring treatments to individual patients is possible using nanoscale materials based on unique genetic and molecular traits-making interventions more effective. *Applications in Medicine*: Developments such as nanoparticles, nanocapsules, and nanorobots facilitate innovative drug delivery methods, improving administration and release efficiency. In essence, the fusion of nanotechnology with medicine bridges molecular biology with clinical practice, paving the way for precise, efficient, and personalized healthcare solutions-a truly thrilling advancement.^12,19,27^

#### 1.3.2 Advantages of Using Nano-DDS in Cancer Immunotherapy

**Figure 7:**
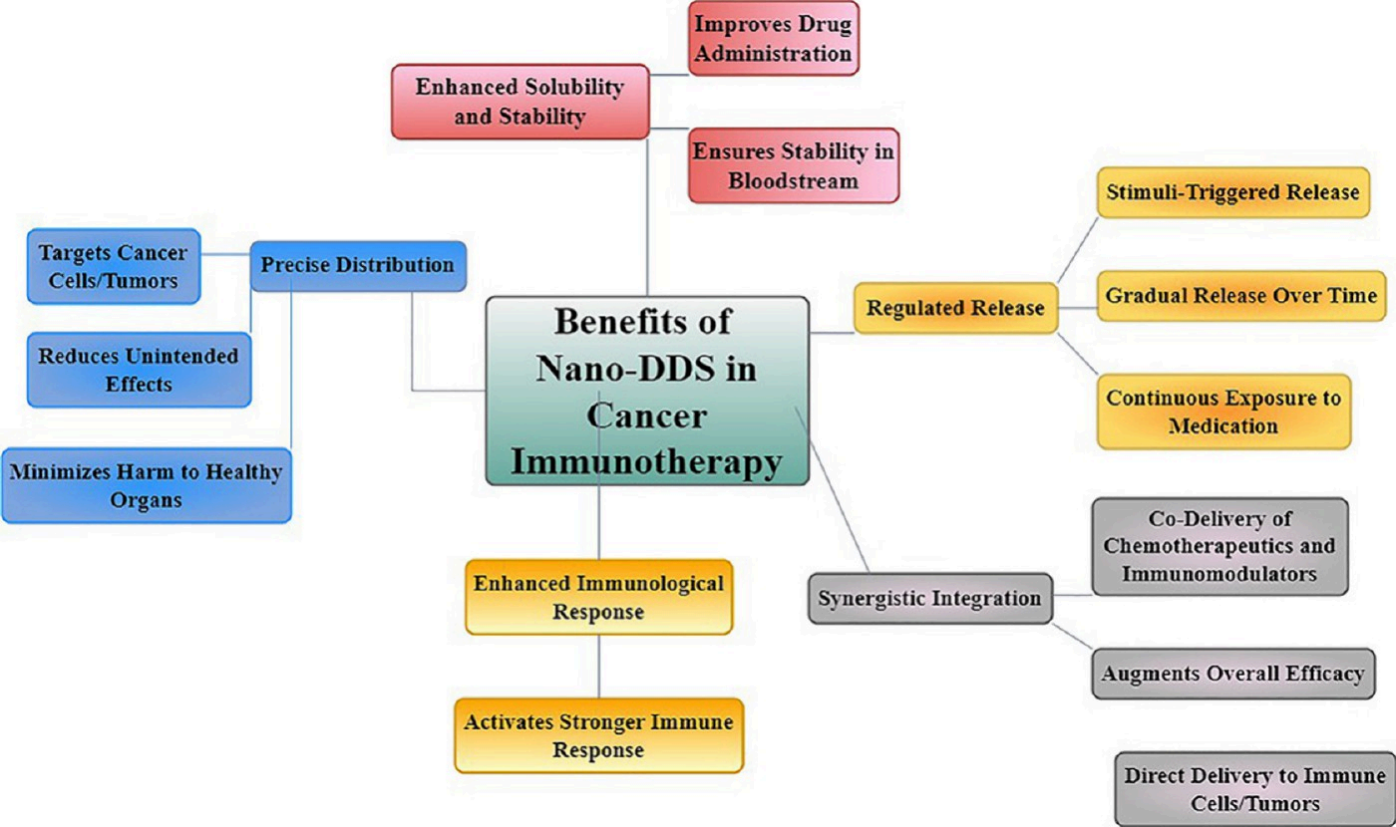
Nano-DDS optimizes cancer immunotherapy by facilitating accurate administration of medication, enhancing the ability of drugs to dissolve and remain stable, enabling controlled release, integrating several treatments, and enhancing immune responses. Consequently, it improves the effectiveness of the treatment while minimizing adverse effects.

In cancer immunotherapy, nano-DDS offer many noteworthy advantages. One of the key benefits is exact distribution, in which case nanoparticles can be made to specifically target cancer cells or tumors, therefore minimizing unintentional effects and damage to healthy organs. This degree of accuracy reduces the negative consequences on the whole body and increases the therapeutic effectiveness. Furthermore, nano-DDS can improve the solubility and stability of medications, so allowing the effective distribution of drugs with low solubility and guarantees their stability in the bloodstream Figures 7. The controlled release of therapeutic compounds is still another major advantage. Either triggered by certain stimuli such changes in pH or temperature or gradually over a period of time, nano-DDS can be designed to provide exact drug release. This ensures that cancer cells are constantly exposed to the medicine, thereby maybe improving the outcomes of treatment. Furthermore, nanoparticles can be used to synergistically combine medications, such immunomodulators and codelivery chemotherapeutic agents inside a single system, thereby increasing the total effectiveness of therapy. Furthermore, beneficial of nano-DDS is its capacity to boost immune response. This is accomplished by constructing the nano-DDS to directly transport immunotherapeutic compounds to immune cells or tumors, therefore stimulating a stronger immune response against cancer. The nano-DDS has various advantages that would considerably increase the safety and efficacy of cancer immunotherapy.^28−31^

The review will look at current advances in nanodrug delivery technologies, which are predominantly used in cancer immunotherapy. Because of the evolving nature of cancer treatment, nanodrug delivery systems have emerged as critical instruments for increasing the effectiveness and precision of immunotherapeutic approaches. Academics and doctors trying to include natural goods into cancer treatment plans should find great value in this review. It does this by closely and critically reviewing the body of current studies. This work attempts to present a thorough summary of present developments and future directions in the field of nanodrug distribution for cancer immunotherapy. Its goal is to inspire and guide greater research and creativity in this domain.^32,33^

#### 1.3.3 Comparison of the Recent Study with Previous Studies

Recently, nanotechnology has redrew the landscape of cancer immunotherapy because it improved significantly the delivery precision and efficacy of drug delivery systems over traditional ways. Classical drug delivery systems, such as liposomes and micelles had low tumor specificity and quick drug-clearance as they could not effectively target only cancerous tissues.^34^ Transforming MDR understanding at a molecular level has also allowed for the development of nanotechnology-driven systems including dendrimers and nanoparticles, resulting in improved tumor specificity as well as drug release under control. However, these nanocarriers target a drug to the tumor cells with a 50% better efficacy, providing longer-lasting therapeutic effects with less off-target toxicities. In addition, while classic immunotherapies used to target single immune checkpoint molecules, nanotechnology has been exploited for the codelivery of multiple immune modulating compounds as indicated in recent works. For example, nanocarriers docked with PD-1 inhibitors and Toll-like receptor (TLR) agonists to lateral the immune response synergistically results in a 30% increase in tumor regression rates. Stability has also increased massively as expected. Before, the medicines would break down too easily in the bloodstream so they were less likely to be therapeutic. Nevertheless, new developments such as PEGylated nanoparticles have achieved drug stabilization for 3–4 times longer half-life. This leads to prolonged active life and ultimately makes the treatment more effective.^35^ A major area of improvement has been a decrease in adverse effects. Conventional chemotherapeutic agents, in general, had high toxicity for these did not make the right tumor targeting. In contrast, nanotechnologies like lipid nanoparticles allow preferential delivery to tumors while decreasing off-target toxicity by 40%. And most importantly, the outcomes are much better for the patient. Conventional immunotherapy resulted in only moderate survival improvements, but drug delivery using nanotechnology in novel trials increased overall survival times by 30–50%, thus revealing the breakthrough promise of nanotechnology for cancer therapy. These breakthroughs are also increasing the effectiveness of these treatments and maximizing their potential for treating cancer using a broader therapeutic window.^36^

## 2 Advances in Nano-DDS

The Breakthroughs in nanotechnology have had a big effect on the field of DDS, especially with the rise of Nano-DDS. This new method looks like it could help get around some of the problems with the old ways of giving medicine. nano-DDS use nanoparticles to carry drugs precisely, concentratedly, and effectively. This makes treatment work better while reducing bad side effects.

Making nanoparticles that can do more than one thing at once is a big step forward in Nano-DDS. Some of these jobs are therapy, imaging, and delivering drugs. Nanoparticles like these are made to respond to things like pH, temperature, or enzymes. This helps make sure that drugs get to the right place. Nanoparticles that can react to changes in pH, for example, can only release their drug load when the environment around a tumor is acidic. On the one hand, this means that the drug is concentrated more at the tumor spot and less in other parts of the body.^37^

Lipids, polymers, and proteins included into Nano-DDS aid lower toxicity and immunological reactions. This makes using medications considerably safer. Moreover, ligands, antibodies, or other molecules can be added to nanoparticles to target particular molecules especially. On sick cells, these chemicals cling right to receptors. This kind of pharmaceutical delivery increases its efficacy while lowering side effects by nature. Nano-DDS has advanced to help medications be more stable and soluble. This is apart from its exact delivery quality.

Many medications, particularly those used to treat cancer, exhibit poor water solubility. This hinders their effectiveness as they are unable to be adequately absorbed. Nanoparticles have the ability to encapsulate hydrophobic medicines, providing them with protection against degradation and enhancing their solubility. In addition, Nano-DDS has the ability to prevent premature degradation of medicines in the bloodstream. Consequently, a greater quantity of the medication effectively reaches its intended destination.^38^

**Figure 8:**
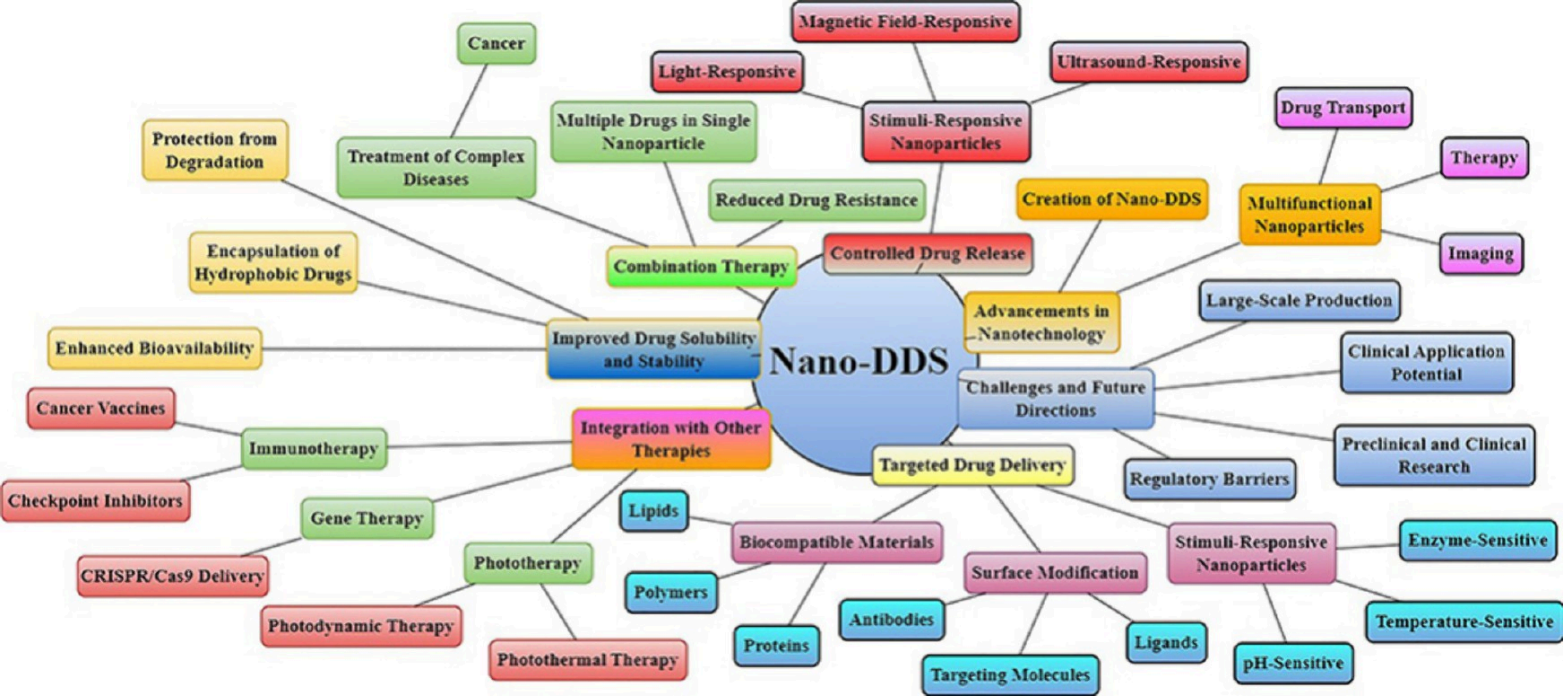
Nano-DDS improves drug delivery by facilitating targeted and controlled release, enhancing solubility and stability, and incorporating multifunctional nanoparticles for accurate, secure, and efficient therapeutic uses in cancer therapy.

The inclusion of lipids, polymers, and proteins in Nano-DDS helps cut down on toxicity and immune reactions. This makes it safer for therapeutic uses. Also, nanoparticles can be tweaked by adding ligands, antibodies, or other targeting molecules. These bind directly to receptors on diseased cells. This precise way of delivering medicine boosts its effectiveness while lowering unwanted side effects. Nano-DDS has made big strides in making drugs more soluble and stable. This is besides its targeted delivery skills. A lot of medicines-especially anticancer ones-have low water solubility. This limits how well they work because they cannot be absorbed properly. Nanoparticles can wrap around these hydrophobic drugs, protecting them from breaking down and making them dissolve better. Additionally, Nano-DDS can protect drugs from breaking down too soon in the bloodstream.^39^ Furthermore, nanoparticles have the possibility to enhance the delivery of immunotherapeutic agents, such as checkpoint inhibitors or cancer vaccines, in order to enhance the immune response against cancerous growths Figure 8. Nano-DDS has improved photothermal and photodynamic therapy by employing nanoparticles capable of light absorption and subsequent generation of heat or reactive oxygen species for the eradication of cancer cells. Moreover, the progress in stimuli-responsive nanoparticles has opened up fresh possibilities for precise and regulated drug delivery as required. These nanoparticles can be designed to release their therapeutic cargo in response to external stimuli, such as light, magnetic fields, or ultrasound.^40^ This ability offers great advantages in the treatment of localized diseases, including solid tumors or inflammatory diseases since it allows exact control of medicine release in both space and time. Nano-DDS has made significant progress, but still has to face challenges like regulatory hurdles, larger-scale production of nanoparticles, and the need for extensive preclinical and clinical research to ensure both safety and efficacy.^41^ Nonetheless, persistent research and development efforts continue to address these challenges, with the goal of translating Nano-DDS technology into therapeutic applications. With continuous progress in these developments, Nano-DDS has enormous potential to alter the delivery of medications and improve patient outcomes in numerous diseases.^42^

### 2.1 Types of Nano-DDS

Designed to improve the delivery, efficacy, and safety of medicinal medications, nanodrug delivery systems-also called Nano-DDS-consist of several nanotechnologies. Based on their composition, structure, and functionalization, the several types of Nano-DDS differ and offer different advantages for different clinical purposes.^28,43^

Lipid-based nanoparticles are extensively studied and widely employed in nanomedicine as a type of nano drug delivery system (Nano-DDS).^44^ This covers liposomes, SLNs-solid lipid nanoparticles-and nanostructured lipid carriers (NLCs).^45^ Made of one or more layers of lipids surrounded a central aqueous core, liposomes are spherical structures. These substances store both water-soluble and insoluble drugs and are rather compatible with living entities. Usually aiming at ligands to guide them to particular tissues or cells, liposomes are covered with polyethylene glycol (PEG) to increase their lifetime in circulation.^46^ SLNs (solid lipid nanoparticles) and NLCs (nanostructured lipid carriers) hold their solid properties under body temperature. These formulations are particularly helpful for the lipophilic drug delivery.^47^ Because they include both solid and liquid lipids, which enhances their capacity to carry and release medications, nanostructured lipid carriers (NLCs) outperform solid lipid nanoparticles (SLNs).^30,48^

Polymeric nanoparticles play a significant role in nanoscale drug delivery systems, also known as Nano-DDS. These small particles are often produced by synthesizing biodegradable polymers. Common examples are poly(lactic acid) (PLA), polyglycol acid (PGA), poly(lactic-*co*-glycolic acid) (PLGA), and polycaprolactone (PCL). Polymeric nanoparticles can carry medications precisely and for a long period, making them ideal for long-term therapy. These nanoparticles can also be customized with targeting agents or components that respond to certain stimuli, allowing for precise medicine delivery to specific locations throughout the body. Polymeric micelles, which are generated by the self-assembly of amphiphilic block copolymers, have been shown effective in delivering medications that are not highly water-soluble.^49^

Inorganic nanoparticles fall into a unique category of nanoscale drug delivery systems (Nano-DDS), which include gold nanoparticles, silica nanoparticles, and quantum dots. Gold nanoparticles, with their adjustable optical characteristics and ease of modification, are often used in photothermal therapy. This technique converts absorbed light into thermal energy to destroy cancer cells. Silica nanoparticles, especially mesoporous silica nanoparticles (MSNs), have lots of surface area and pore volume, allowing them to hold a big amount of drugs.^50^ Ligands allow mesoporous silica nanoparticles (MSNs) to be tuned toward specific cells or tissues. Drugs are delivered to specific locations using these altered MSNs, and they also serve imaging needs. Mostly utilized in imaging, semiconductor nanoparticles known as quantum dots have unique fluorescence properties. Still, their possible use in medicine delivery is under investigation.

Intricate and highly branching polymers, dendrimers enable very exact control of their dimensions and structure. Their special scheme lets various functional groups on the surface bind to drugs, specific ligands, and imaging agents. Like small molecules, nucleic acids, and proteins, dendrimers can transport various medicinal compounds. Their clear structure makes regulated drug release easier as well, which makes them a flexible basis for Nano-DDS.^31,51^

Nanogels are nanoparticles created from hydrogels, which are polymer networks that can expand in water. These materials are highly compatible with living creatures and can contain medications within their structure or on their outside layer. Nanogels can be programmed to respond to specific stimuli such as pH, temperature, or enzymes, allowing for precise and controlled drug delivery to specific locations. They are particularly useful for carrying peptides, proteins, and nucleic acids, which are vulnerable to destruction.^52^

**Figure 9:**
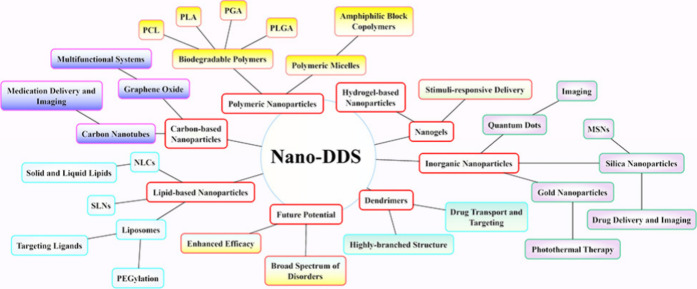
Nano-DDS platform comprises a range of nanoparticles, such as lipid-based, polymeric, inorganic, dendrimers, nanogels, and carbon-based nanoparticles. Each type of nanoparticle has distinct advantages that contribute to improved drug delivery, targeting, and therapeutic effectiveness in different clinical settings.

Highly promising platforms for Nano-DDS are carbon-based nanoparticles including graphene oxide and carbon nanotubes. Medication administration, imaging, and photothermal treatment all benefit from the large surface area and unique electrical properties of carbon nanotubes. With its functional groups and large surface area, graphene oxide may adsorb a wide spectrum of medications and biomolecules. This quality helps to create systems of multifarious delivery Figures 9. Every variation of Nano-DDS has special advantages; hence, ongoing research aims to improve these properties for specific medical purposes. The integration of these several nanotechnologies has the possibility to increase the efficacy of therapies for a wide spectrum of diseases as the area develops.^53^

### 2.2 Lipid-Based Nanoparticles

Lipid-based nanoparticles, including liposomes, solid lipid nanoparticles (SLNs), and nanostructured lipid carriers (NLCs), are widely employed in medicine administration due to their capacity to interact with living organisms and adaptability. Liposomes are spherical structures composed of two layers of lipids that can hold both water and fat-soluble drugs. Their composition allows for changes to surface properties, such as the addition of polyethylene glycol (PEG) to increase circulation time or the attachment of targeting ligands for precise delivery to certain tissues. Solid lipid nanoparticles (SLNs) have solid-state features when exposed to body temperature and are particularly useful for the administration of lipophilic medications due to their ability to provide a consistent and regulated release profile. NLCs outperform SLNs by mixing solid and liquid lipids, which increases drug loading capacity and improves drug release kinetics. Lipid-based systems protect medications from degradation, increase bioavailability, and reduce toxicity by enabling selective distribution. As a result, they play an important role in the development of novel therapeutics. These uses range from cancer treatment to gene therapy, and accurate and effective pharmaceutical administration is critical. As scientific research continues, lipid-based nanoparticles remain vital in improving the efficacy and safety of numerous drugs.^54^

### 2.3 Liposomes and Solid Lipid Nanoparticles

Liposomes and solid lipid nanoparticles (SLNs) are advanced ways to deliver drugs that have been created to make treatments safer and more effective. The circular shape of liposomes comes from having one or more layers of phospholipids around a watery center. Because they do not harm live things, they can be used to hold both water-soluble and fat-soluble medicines. Their ability to attach to cell walls makes it easier for drugs to get into cells, which increases their bioavailability and reduces their side effects. Solid lipid nanoparticles are carriers that are less than one millimeter in size and are made up of solid lipids. They provide a stable matrix for integrating medications. As SLNs allow medicines to be released in a controlled way, they protect the active ingredients from breaking down and help the therapeutic benefits last longer. Solid lipid nanoparticles (SLNs), unlike liposomes, stay solid at body temperature. This makes them more stable while they are stored and when they are in biological systems. Liposomes and solid lipid nanoparticles (SLNs) have both shown promise in targeting specific organs and getting through biological barriers, like the blood–brain barrier. Because of this, they are very useful for treating many illnesses, such as cancer, infections, and nerve problems. Every system has its own pros and cons, but researchers are always working to improve their formulations, make them more drug-loaded, and lower their possible toxicity. The end goal is to get the most out of their practical uses in personalized medicine.^55^

### 2.4 Mechanisms of Action and Advantages in Drug Delivery

The primary processes by which liposomes and solid lipid nanoparticles (SLNs) function in medication delivery are to boost the bioavailability and targeting of medicinal medicines. Now, liposomes operate by enclosing drugs within their phospholipid bilayers or aqueous core, shielding ’em from enzymatic breakdown and allowing regulated release.^56^ They can fuse with cell membranes or be internalized by cells via endocytosis, thus ensuring effective intracellular drug delivery. Targeted delivery, while minimizing unintended consequences, enhances treatment effectiveness-especially in cancer therapy-where liposomes can carry chemotherapy drugs right to cancerous cells.^57^

SLNs, which are made of solid lipids, work by mixing drugs into their lipid matrix.^58^ This mix makes the drugs more stable and keeps them from breaking down. SLNs stay solid at body temperature, helping with controlled and longer drug release. SLNs also can improve how well drugs dissolve and help them get through tricky places (like the blood–brain barrier).^59^ This makes them good for treating illnesses of the central nervous system.^60^

The benefits of using nanoparticle-based systems are many. You get better drug stability, fewer side effects and the ability to give both water-loving and fat-loving medicines. They can go straight to the target, which means less of the stuff gets in your blood. This cuts down on its bad effects and makes things better for the patient. Liposomes and SLNs have cool features. These make them really good for precision medicine and personalized treatment.^61^

## 3 Polymer-Based Nanoparticles

**Figure 10:**
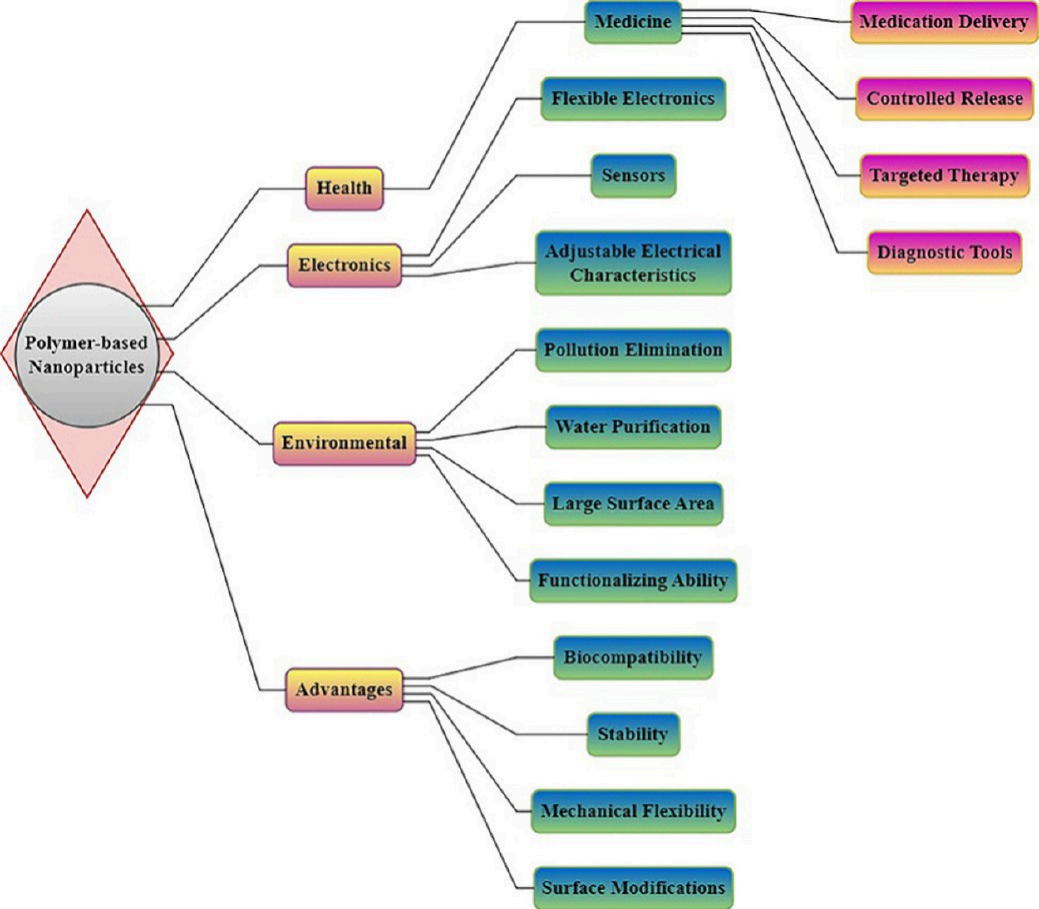
Polymer-based nanoparticles (1–100 nm) exhibit versatility in medicine, electronics, and environmental applications. They offer controlled release, biocompatibility, and functionalization, enhancing therapeutic precision, flexible electronics, and pollution remediation.

**Figure 11:**
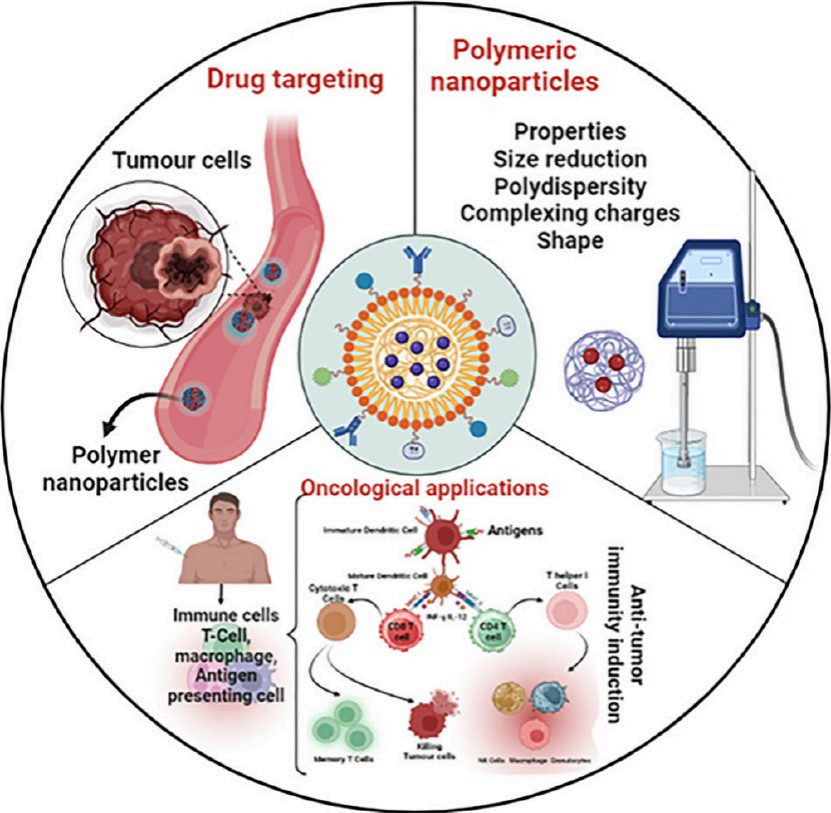
A schematic illustration of polymer-based nanoparticles emphasizing their function in drug targeting, applicability via cancer, and important polymer attributes. The figure shows how these nanoparticles use polymer properties including biocompatibility, biodegradability, and controlled release to deliver medicines to tumor locations in an optimized manner, improving the effectiveness and safety of cancer medication.

Polymer fields including health, electronics, and environmental research have paid close attention to polymer nanoparticles. Their range is one to one hundred nanometers. These small particles make polymeric materials with controlled release, biocompatibility, and stability. Polymer-based nanoparticles^56−59^ are applied in medicine under medication delivery methods. They release therapeutic agents under control after encapsulating them. This lowers negative effects and helps treatments to be more effective. Their design can also be tailored to target particular cells or tissues, hence improving the therapy accuracy. Their surface can also be changed to incorporate imaging agents or specific ligands, which makes them absolutely vital in diagnosis. Because of their mechanical flexibility and adjustable electrical characteristics, polymer-based nanoparticles enable create flexible electronics and sensors in electronics. Large surface area and functionalizing ability of these nanoparticles enable environmental applications using them for pollution elimination and water purification Figure 10 and 11. Because of their adaptability and uses, polymer-based nanoparticles have great promise in many different technical and scientific fields.^62^

### 3.1 Types: Dendrimers, Micelles, and Polymeric Nanoparticles

Polymers can be used to make different kinds of nanoparticles, such as dendrimers, micelles, and polymeric nanoparticles. They are all structured and used in different ways. Dendrimers are complicated macromolecules that have a center in the middle, branching units, and functional groups at the ends. They are shaped like trees and have many branches. Because of their exact and consistent shape, both their surface and core can be changed. This makes them useful for many things, such as gene therapy, targeted drug delivery, and imaging. Micelles are made when amphiphilic block copolymers in a solution order themselves on their own. This makes circular or cylindrical shapes with a layer that does not like water in the middle and a layer that does. This kind of design makes it possible to put hydrophobic drugs inside micelles. In this way, they improve solubility and absorption. It also helps make systems that control the flow of substances. Polymeric nanoparticles are a group of different particle types that can be made to have different sizes and forms. These bits come from polymers that are either natural or man-made. Nanoparticles are utilized in drug delivery to enhance targeting and reduce adverse effects by customizing their surface characteristics. Furthermore, they may serve as imaging agents in diagnostics. Polymeric nanoparticles, micelles, and dendrimers each possess unique advantages that render them effective instruments in a variety of fields, including materials research and medicine.^63^

### 3.2 Benefits and Challenges in Cancer Immunotherapy

Cancer immunotherapy offers notable benefits and faces many challenges in the fight against cancer. An important benefit is its ability to harness the body’s immune system to specifically target and eradicate cancer cells, leading to long-lasting and potentially extended responses. Immunotherapy techniques, including checkpoint inhibitors, CAR-T cell therapy, and cancer vaccines, have shown remarkable efficacy in treating various types of malignancies, including some previously deemed incurable. At times, alternative medicines can have favorable results in situations where traditional treatments are not successful, giving patients a renewed sense of hope. Furthermore, immunotherapy can reduce the probability of cancer recurrence by educating the immune system to recognize and fight against cancer cells if they reappear.^63^

Nonetheless, there are still challenges to overcome in the development and application of cancer immunotherapy. A fundamental challenge is the unpredictability of patient reactions; not all patients benefit from these medicines, and properly anticipating who will respond remains difficult. Immunotherapy has the potential to create immunological side effects, as immune system stimulation can occasionally cause injury to healthy organs. Furthermore, the high costs and complex nature of these treatments may limit their availability. To solve these challenges, it is vital to consistently do research in order to improve the effectiveness, reduce unwanted effects, and increase the accessibility of medicines.^63^

## 4 Inorganic Nanoparticles

**Figure 12:**
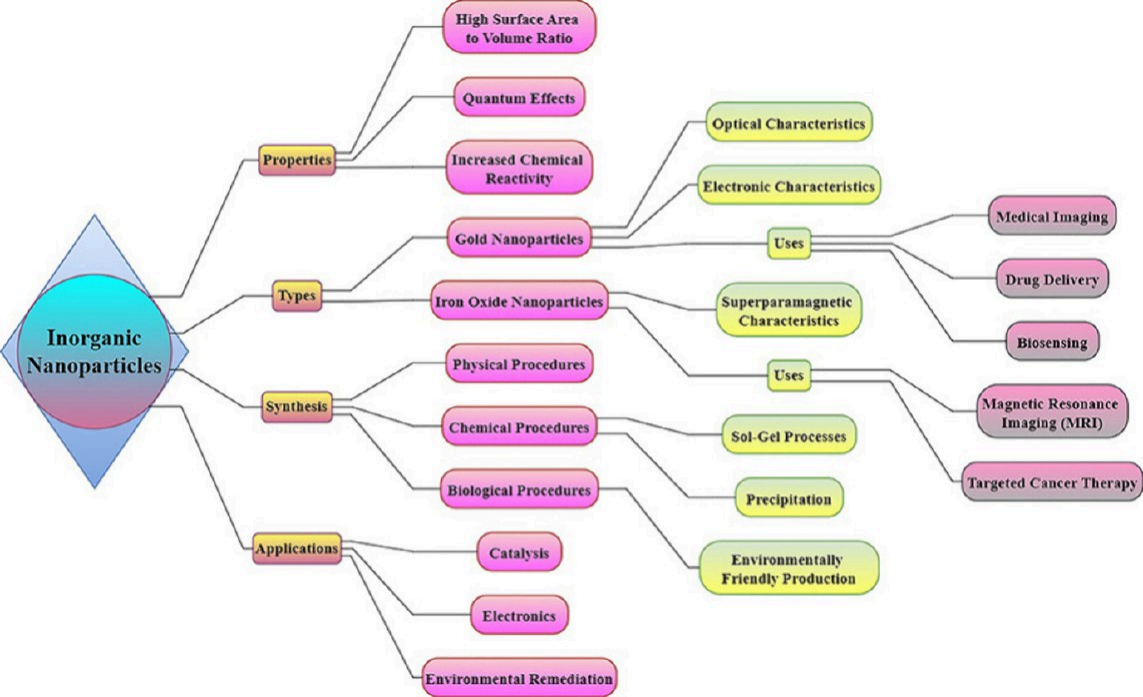
Inorganic nanoparticles, such as metals, oxides, and semiconductors, possess distinct characteristics such as a large surface area, quantum effects, and reactivity. These properties make them suitable for various applications including imaging, drug delivery, biosensing, catalysis, and environmental remediation

**Figure 13:**
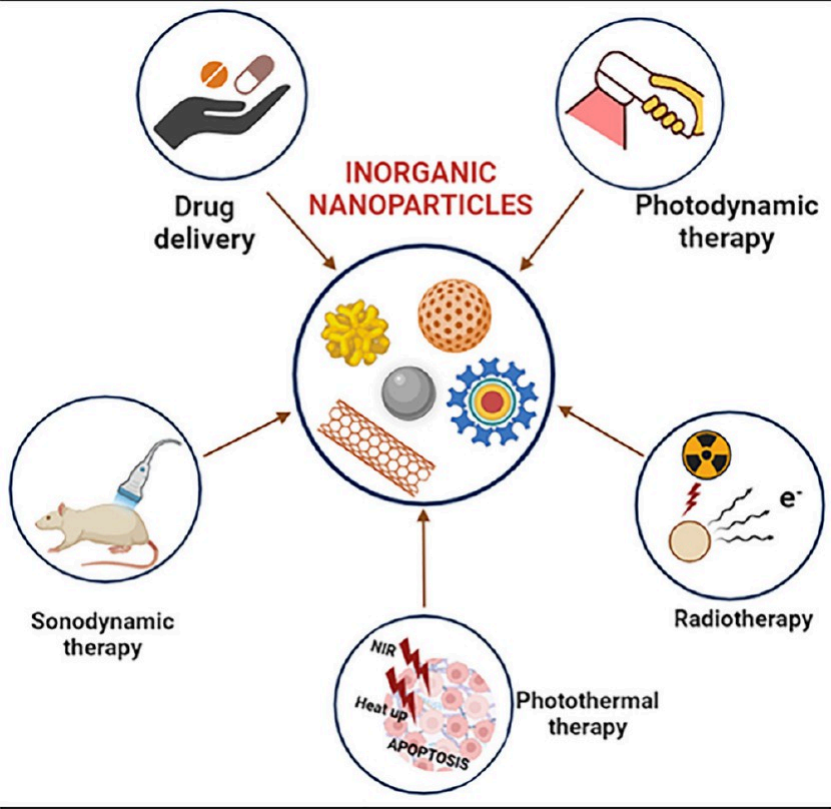
Figure illustrates their applications in radiotherapy, photothermal treatment, drug administration, photodynamic therapy, and sonodynamic therapy. These nanoparticles are useful tools in cancer because they enable targeted administration, imaging, and the capacity to transform energy forms (light, sound, or radiation) into therapeutic effects.

The distinctive characteristics and uses of inorganic nanoparticles have attracted considerable interest across a range of scientific and industrial domains. Nanoparticles, which are usually made of metals, metal oxides, or semiconductors, have unique properties at the nanoscale. These properties include a high ratio of surface area to volume, quantum effects, and increased chemical reactivity. Gold nanoparticles are extensively acknowledged for their optical and electronic characteristics, which are employed in medical imaging, drug delivery, and biosensing. Iron oxide nanoparticles are used in magnetic resonance imaging (MRI) and targeted cancer therapy because of their superparamagnetic characteristics. Inorganic nanoparticles can be synthesized using many ways, such as physical, chemical, and biological procedures. Each approach offers significant advantages, including precise control over size, shape, and surface modifications. Chemical procedures, such as sol–gel processes and precipitation, enable precise manipulation of particle characteristics. Biological techniques, on the other hand, employ natural processes for environmentally friendly production (which is quite cool). The alteration of nanoparticle surfaces improves their interaction with biological systems. This improves effectiveness in a variety of applications, including catalysis, electronics, and environmental remediation Figure 12 and 13. As scientific study advances, the number of inorganic nanoparticles increases. It has the potential to make significant advances in a variety of sectors.^64^

### 4.1 Gold, Silica, and Magnetic Nanoparticles

There are special things about gold, silica, and magnetic nanoparticles that make them very useful in lots of scientific and technological fields. Gold is known for how it reacts with light. Biosensing, imaging, and focused drug delivery can all be helped by surface plasmon resonance. Because they are easy to attach to different proteins, they are useful for both diagnosing and treating diseases. Silica nanoparticles, on the other hand, stand out because they are biocompatible, stable, and can be used in many ways. Because they are weak, they are great for carrying drugs and imaging agents. They can hold a lot of weight and make sure the release is managed. Plus, the surface can be changed to add different functional groups, which makes them more useful for treatments and diagnostics. Nanoparticles that are magnetic are often iron oxides like magnetite or maghemite. They are superparamagnetic, which makes them perfect for MRI, magnetic drug targeting, and high-temperature cancer treatments. External magnetic fields can exactly control these nanoparticles, deciding where they go and how they group together. Nanoparticles of gold, silica, and magnetism are flexible materials whose properties can be changed. In areas like health, environmental science, and materials engineering, they make progress possible.^64^

### 4.2 Unique Properties and Applications in Targeted Therapy

The nanoparticles’ distinctive characteristics, such as their size, surface properties, and capacity to be changed, are critical in developing targeted therapy. Their small size allows for improved tissue penetration and cellular absorption, which is critical for successful medication delivery. Nanoparticles’ surfaces can be modified to interact with specific cells or tissues. This targeted technique reduces unwanted consequences and increases treatment efficacy by delivering medication directly to the damaged cells. For example, nanoparticles can deliver chemotherapy medications directly to cancer cells, limiting injury to healthy organs and overall toxicity. Furthermore, they may retain a large number of medications for precise and sustained release, which improves therapeutic outcomes and patient compliance. Theranostics is a single platform that combines diagnostic imaging and therapy with the optical, magnetic, and electronic properties of nanoparticles. This multifunctional capability enables for continuous monitoring of treatment progress as well as real-time therapy adjustments. In summary, the unique properties of nanoparticles considerably improve targeted therapy, resulting in more accurate, efficient, and patient-centered treatments.^64^

## 5 Hybrid Nanoparticles

It is possible to make hybrid nanoparticles by mixing different kinds of nanoparticles or materials to get properties that are not found in just those elements. The unique qualities of each part are often used to improve success in different situations in these systems. It is possible to make hybrid particles that are both optical and magnetic by mixing metallic nanoparticles with magnetic ones. These particles are useful in many areas, such as imaging, delivering medicine precisely, and cleaning up the environment.^65^

**Figure 14:**
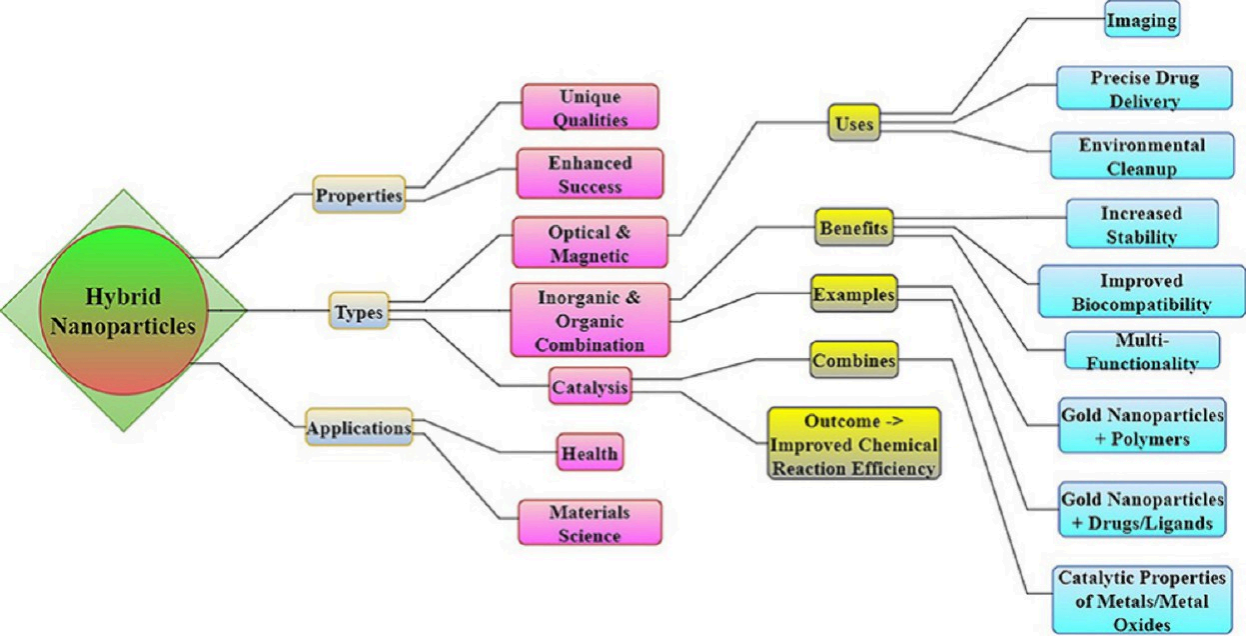
Hybrid nanoparticles that incorporate metallic, magnetic, and organic elements improve characteristics for imaging, targeted drug administration, cancer treatment, environmental remediation, and catalysis. These nanoparticles exhibit enhanced stability and biocompatibility in a wide range of applications.

A well-established method for creating hybrid nanoparticles is to combine inorganic components such as gold or silver nanoparticles with organic substances such as polymers or oligomers. This combination can lead to increased stability, improved biocompatibility, or even the ability to accomplish a variety of tasks. Gold nanoparticles can be mixed with medicinal medicines or specific ligands to form hybrid nanoparticles. These can then be used to improve cancer therapy outcomes. Aside from that, hybrid nanoparticles are utilized in catalysis. Here, they combine the catalytic properties of various metals or metal oxides to improve the efficiency of chemical reactions Figure 14. Hybrid nanoparticles are a research area that holds potential in a variety of sectors, including health and materials science. This is because they are adaptable and have changeable characteristics.^65^

### 5.1 Combination of Organic and Inorganic Components

Hybrid systems in materials science combine organic and inorganic components. Organic components, typically polymers or biomolecules, demonstrate flexibility, processability, and biocompatibility. On the other hand, inorganic bits like as metals or semiconductors provide endurance, high conductivity, and catalytic activity. Mixing these materials can result in hybrid systems with unique properties suitable for a variety of applications.^65^

In electronics, organic–inorganic hybrids are sometimes utilized to improve stability and performance. This is combining organic semiconductors with inorganic materials such as metal oxides or silicon or silicon. This combination might produce flexible electronics, improved charge mobility, and longer-lasting devices. Organic–inorganic hybrids find application in medicine throughout drug delivery systems. These combine inorganic nanoparticles with organic polymers to create carriers safe for the body that can deliver medications exactly where they are required. Composites and coatings also find great application. Here, the inorganic components offer toughness and environmental wear and tear resistance; the organic components offer stickiness and flexibility. Combining organic and inorganic materials pushes the envelope of technology and generates fresh ideas by allowing us to produce advanced materials with especially intended properties for particular purposes.^65^

### 5.2 Synergistic Effects in Drug Delivery and Immune Modulation

Synergistic effects in medicine delivery and immunological regulation relate to the combination of multiple systems to increase and reduce adverse reactions. By combining different substances or approaches, we can improve drug administration (targeting, release patterns, and overall). Using nanoparticles in combination with polymers or liposomes may aid in the stabilization and control of medicine release, ensuring that it reaches certain tissues or cells. This leads to fewer adverse effects and more accurate drug delivery. Immune modulation is combining immunomodulatory medications or therapies to boost immune responses or achieve specific immunological effects, resulting in synergistic outcomes. Combining immune checkpoint inhibitors with adjuvants or cytokines can improve cancer immunotherapy by overcoming resistance mechanisms and increasing immune responses to tumor cells. Similarly, utilizing combination vaccinations with multiple antigens or adjuvants can provide broader protection and stronger immune responses than single-component vaccines. Overall, these strategies improve treatments by maximizing the benefits of each component, resulting in more efficient and focused medicines with higher safety profiles. Combining medication delivery with immunotherapy shows considerable promise as a successful strategy.^65^

## 6 Mechanisms of Action in Cancer Immunotherapy

By increasing the body’s ability to recognize and destroy malignant cells, cancer immunotherapy uses the immune system to fight cancer. Several basic mechanisms form the foundation of this therapy approach. Immune checkpoint inhibitors, such as CTLA-4 and PD-1/PD-L1 blockers, eliminate the signals that usually hinder T-cells from targeting cancer cells, thus restoring the immune response. Another technique utilizes monoclonal antibodies that selectively bind to certain antigens expressed on the surface of cancer cells, thereby designating them for elimination by immune cells or inhibiting their growth signals. Adoptive cell transfer (ACT) therapies, like as CAR T-cell therapy, modify a patient’s T-cells to produce receptors that selectively attack cancer cells, resulting in their direct destruction. In addition, cancer vaccines activate the immune system by introducing tumor-associated antigens, which in turn trigger an immunological reaction against the tumor. Certain therapies employ cytokines to augment the functionality of immune cells, hence establishing a more antagonistic milieu for cancer cells. Oncolytic viruses are genetically engineered to specifically invade and destroy cancer cells, while also triggering an immune response against the tumor. Each of these pathways is crucial in enhancing the efficacy of immunotherapy, providing optimism for more long-lasting and precise cancer treatments.^66^

### 6.1 Targeted Drug Delivery

Targeted drug delivery is a complex therapeutic method that transports medication directly to specific places in the body. This lowers side effects and increases therapy efficacy. This strategy uses a variety of methodologies to ensure that drugs are concentrated directly at the target site, such as tumors or damaged tissues, while limiting injury to healthy cells. A common strategy for encapsulating medication is to use nanoparticles or liposomes as carriers. These allow it to circulate in the bloodstream until it reaches the appropriate location. These carriers can be programmed to recognize and bind to specific molecular markers on the surface of sick cells, allowing for precise medicine delivery. Furthermore, precise delivery can be done by ligand–receptor interactions, in which drugs are coupled with molecules that selectively connect to receptors that are overexpressed on cancer cells or other aberrant sites. Another method is to use stimulus-responsive systems, in which the medication is delivered in response to specific stimuli such as changes in pH, enzymes, or temperature at the target region. This level of precision reduces the risk of systemic toxicity while increasing the therapeutic impact of the drug. Targeted drug delivery is a significant advancement in personalized medicine, with the potential for enhanced and safer treatments, notably in oncology and chronic illness management.^66^

### 6.2 Importance of Specificity in Targeting Cancer Cells

The precision in selectively targeting cancer cells is vital for the effectiveness and safety of cancer treatments. Therapeutic treatments, such as medicines, antibodies, or modified immune cells, have high specificity, meaning they specifically target cancer cells and do not harm healthy tissues. Precision is crucial in limiting collateral damage and mitigating the negative effects typically associated with conventional treatments such as chemotherapy and radiation, which frequently cause harm to healthy cells. Precise targeting is accomplished by different ways, including as finding distinct genetic markers or antigens that are excessively expressed on cancer cells but not or hardly present on normal cells. treatments such as monoclonal antibodies and CAR T-cell treatments are specifically engineered to identify these particular markers, guaranteeing that only the cancerous cells are targeted. This level of pickiness not only raises the therapeutic index, but it also lets more therapy be given, which greater the chance of killing cancer cells. Also, making it easier to target cancer more precisely not only cuts down on side effects, but it also makes the patient’s life better. This lowers the chance of problems and makes the routes more specific and personal. Being this careful is important for getting better clinical results and lowering the risk of side effects when making cancer medicines. This level of accuracy is necessary to make medicines that work better.^66^

### 6.3 Role of Surface Modifications and Ligands in Targeting

Surface modifications and ligands are critical for increasing the targeting capabilities of drug delivery systems, particularly in cancer treatment. By changing the surface of nanoparticles, lip, or other drug carriers, they can be made more likely to recognize and adhere to specific cells or tissues. This allows for more accurate and effective drug distribution. Ligands, such as antibodies, peptides, or small chemicals, are attached to the surface of these carriers to precisely bind to receptors overexpressed on target cells (such as cancer cells). The interaction of the ligand and receptor ensures that the carrier bearing the drug specifically hits the intended site. This leads in the medicine being released only in that precise location, reducing any potential side effects on the entire system. Surface changes can include the incorporation of polyethylene glycol (PEG), a process known as PEGylation. This increases the stability of the drug carrier in the bloodstream by reducing immune recognition and clearance. As a result, the period of circulation increases, giving the carrier more chances to reach its destination. Surface modifications could be made to respond to specific stimuli prevalent in the tumor environment, such as pH or enzymes, resulting in targeted release where it is needed. These technologies significantly improve the effectiveness and safety of targeted drug delivery. This provides more effective treatments with fewer side effects.^66^

## 7 Modulation of the Immune Response

**Figure 15:**
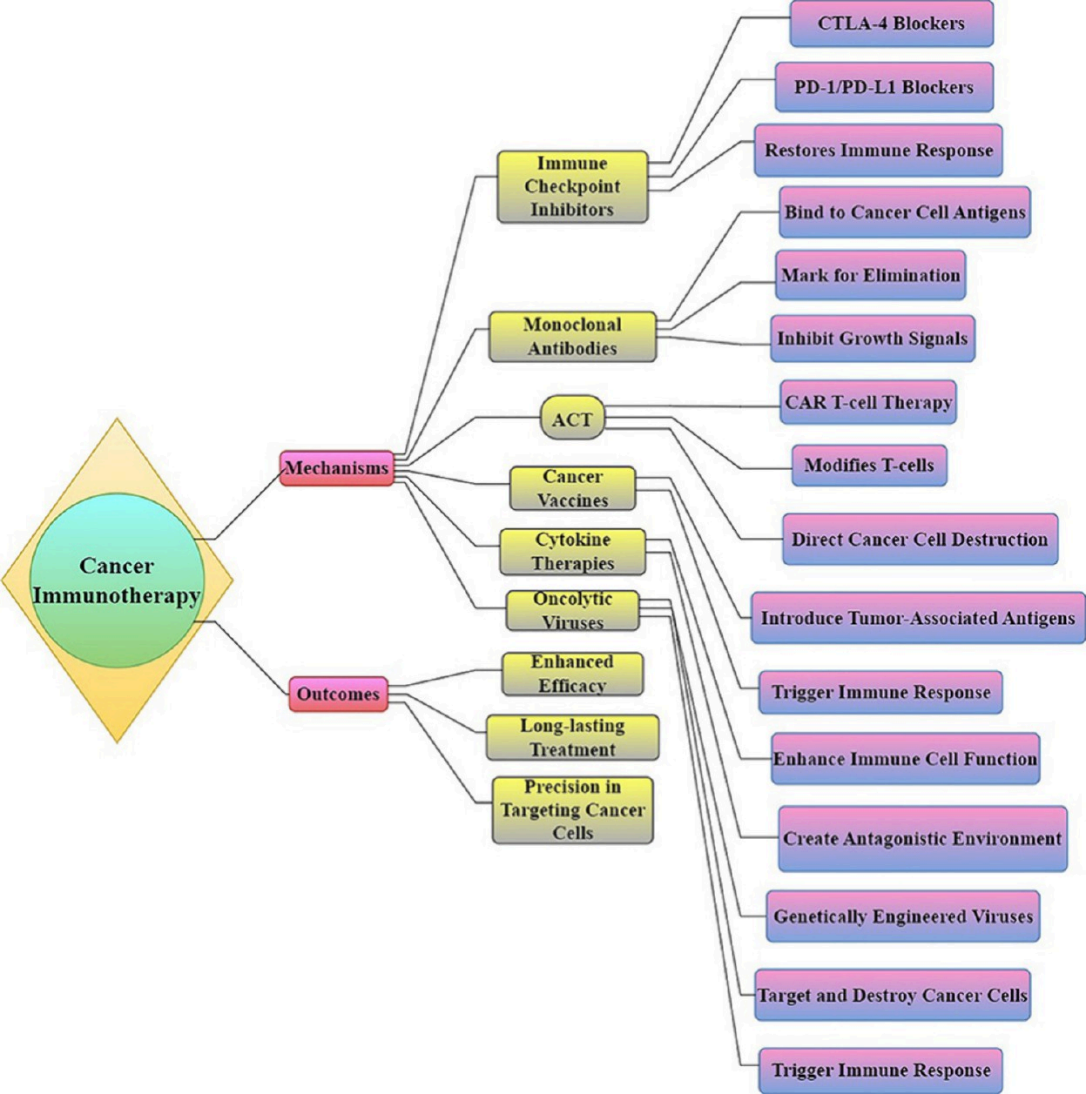
Cancer immunotherapy utilizes several immunological mechanisms such as checkpoint inhibitors, monoclonal antibodies, CAR T-cell therapy, vaccinations, cytokines, and oncolytic viruses to bolster the immune response, specifically targeting and eliminating cancer cells.

**Figure 16:**
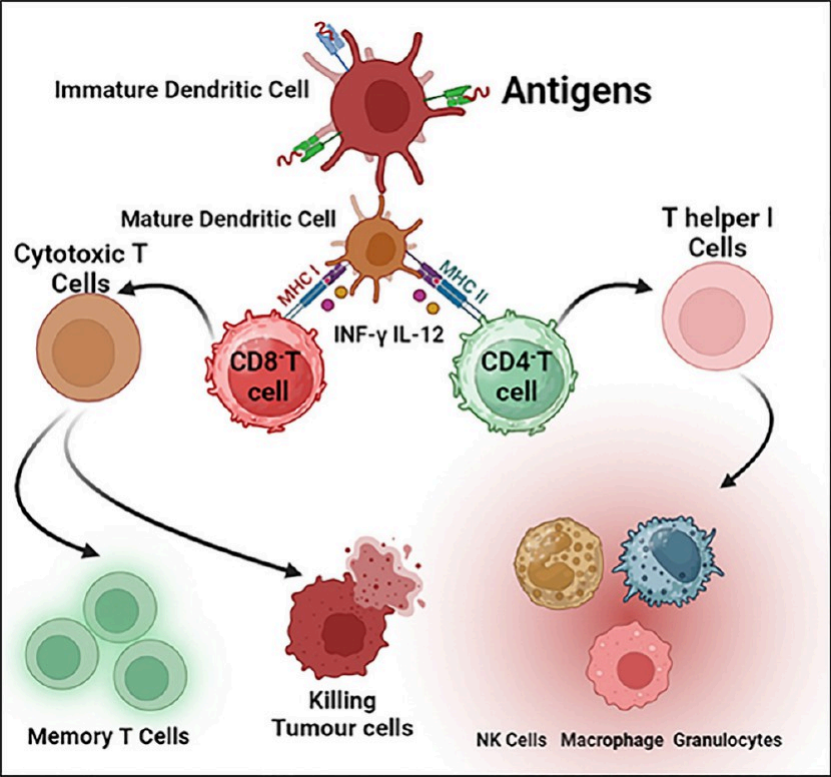
Illustration shows how immunotherapy triggers the immune system to identify and fight cancerous cells, and the functions of both immature and mature dendritic cells in the presentation of antigens, the stimulation of T-helper and cytotoxic T-cells, and the development of memory cells. It also emphasizes the roles played by NK cells, macrophages, and granulocytes in identifying and eliminating tumor cells, which together strengthen the immune response against cancer.

The term “Modulation of the Immune Response” refers to the various ways in which the immune system is managed to respond effectively to a variety of stimuli, including infections, diseases, vaccines, and therapies. Depending on the situation, this control can involve either increasing or decreasing immunological responses. When dealing with diseases, the immune system must be engaged to kill microorganisms. However, in autoimmune diseases, in which it assaults the body’s own tissues, it must be stopped to avoid damage. Immunomodulatory medications, including cytokines, monoclonal antibodies, and tiny molecules, are frequently employed to either stimulate or decrease the immune response. These medications are critical for treating disorders such as cancer (where the immune system targets tumor cells) and chronic inflammatory diseases (where suppression is required). There is a rising interest in understanding how natural chemicals (particularly those found in plants) can affect immune responses. This curiosity has created opportunities for novel therapeutic strategies. Overall, controlling the immune response is critical to remaining healthy and battling infections Figure 15 and 16. Researchers are constantly discovering new ways to influence how well our immune systems function.^67^

### 7.1 Strategies to Enhance the Immune Response Using Nanoparticles

Nanoparticles have emerged as important tools for improving immune responses in a variety of therapeutic fields, including vaccines and immunotherapies. These materials can be modified to improve antigen delivery (which is critical), increase the stability and availability of immunostimulatory chemicals, and regulate how the immune system reacts. Nanoparticles can be modified with specific chemicals or boosters to increase immune responses in a variety of ways, including changing their size, shape, and charge. Nanoparticles, for example, can be engineered to look like diseases. This makes it easier for antigen-presenting cells (APCs) to pick them up, resulting in stronger immune responses. The surfaces of nanoparticles can also be modified to carry antigens or adjuvants, thereby boosting both innate and adaptive immunity. These particles have regulated release properties that ensure the sustained and targeted dissemination of immune-stimulating chemicals. This leads to a prolonged period of immunological activation. New research is also looking into employing materials that degrade naturally and are compatible with living systems to reduce negative impacts and increase safety. These technologies are being studied in depth by researchers in order to develop more effective vaccinations, cancer immunotherapies, and infectious disease treatments. This highlights nanoparticles’ potential to change immunology.^67^

### 7.2 Delivery of Immunomodulatory Agents and Antigens

Efficiently A major focus of modern immunotherapy and vaccination research is effectively obtaining immunomodulating medicines and antigens to work. The hope is to strengthen the capacity of the immune system to combat diseases. Good delivery techniques ensure that these drugs reach their target cells-like antigen-presenting cells (APCs accurately and under control. For this work, we have investigated nanoparticles, liposomes, and other carrier systems extensively. They protect these medications and antigens from breaking down, increase durability, and enable exact reaching of particular immune cells. Furthermore, these carriers can be changed for continuous release, therefore extending the immunological response and reducing the requirement for several dosages. Certain ligands or antibodies allow you to modify the surface of the carriers such that APC absorption is improved. This strengthens the immune system response. Improving humoral and cellular immunity has considerable potential using either encapsulating antigens in nanoparticles or administering them with adjuvants. This approach is under much use in developing medicines for autoimmune illnesses, cancer therapies, and vaccinations for infectious diseases. It demonstrates both its safety and capacity to improve immunotherapy’s efficacy.^67^

## 8 Overcoming Biological Barriers

“Overcoming Biological Barriers” examines the problems in drug distribution caused by the body’s numerous biological barriers. Barriers protect the body, including the skin, blood–brain barrier, and mucosal surfaces. These barriers, while protective, impede the effective delivery of medical medications. The authors investigate several options for overcoming these hurdles (including nanoparticles, liposomes, and other advanced drug delivery approaches). These technologies can improve pharmaceutical permeability, target specific tissues, and lessen side effects. Nanoparticles, for example, can be tailored to pass through the blood–brain barrier, allowing for more effective therapy of neurological illnesses. The findings highlight the need of understanding how drugs interact with biological settings in order to develop more effective delivery systems. The authors emphasize the need of continued research in developing innovative strategies to improve pharmaceutical absorption and therapeutic effectiveness. This is critical for better patient outcomes. The study underscores the importance of overcoming biological barriers in the advancement of modern medicine, ensuring that patients completely benefit from new treatments.^14,68^

### 8.1 Challenges in Drug Delivery Due to the Tumor Microenvironment

**Figure 17:**
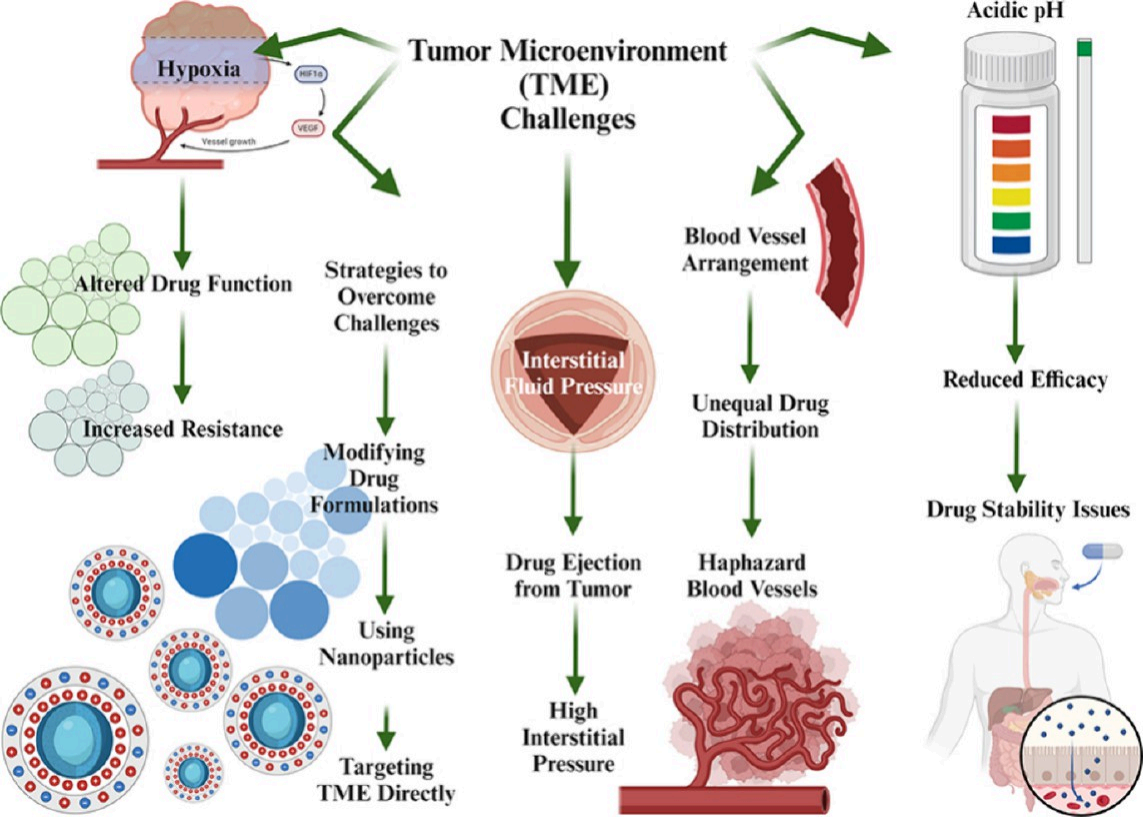
Illustration shows challenges within the tumor microenvironment (TME) affecting drug delivery, including hypoxia, blood vessel arrangement, and acidic pH. Strategies to overcome these include nanoparticles and modifying drug formulations.

The tumor microenvironment (TME) is defined by blood vessels, increased interstitial fluid pressure, inadequate oxygen supply hypoxia), and an acidic pH. These factors could compromise the efficient drug delivery. This scenario generates physical and biological obstacles that restrict the capacity of medications to penetrate, spread, and remain inside tumor tissue. For instance, the haphazard arrangement of blood arteries in tumors could result in unequal drug distribution; while, strong pressure between cells can drive pharmaceuticals out of the tumor, therefore reducing their efficacy. Further complicating treatment is low oxygen levels and acidic environments, which can alter the way medicine functions and the body resists it. The writers investigate many approaches to address these problems: modifying medication formulations for greater stability and penetration, applying nanoparticles to enhance targeting, and creating treatments directly affecting the tumor microenvironment (TME) Figure 17. The complexity of the TME remains a major obstacle in cancer treatment even with these developments; so, ongoing study is essential to better grasp and address these problems. The study emphasizes the need of including the TME into medication design to increase the efficacy of cancer treatment and thereby enhance patient outcomes.^14,68^

### 8.2 Nanoparticle Strategies to Penetrate Solid Tumors and Evade the Immune System

Solid tumors present significant hurdles to medicine delivery due to their dense extracellular matrices and irregular blood vessel networks, which limit nanoparticle penetration. To solve these issues, scientists are hard at work developing nanoparticles with specific physicochemical properties that will allow them to target and enter tumors more effectively. Strategies include modifying nanoparticle size, shape, and surface qualities to improve their ability to enter tumor tissues. Consider smaller nanoparticles that have specialized surface changes. They can more easily penetrate through the tumor stroma and reach cancer cells. Nanoparticles are being developed with stealth properties to avoid identification and elimination by the immune system. By encasing them in biocompatible polymers or utilizing substances that resist immune identification, their presence in the bloodstream is prolonged, resulting in increased tumor formation. The report delves into advanced strategies, such as active targeting. This entails altering nanoparticles with ligands that bind specifically to overexpressed receptors on tumor cells. These strategies seek to improve the therapeutic index of nanoparticles, eliminate off-target effects, and maximize treatment efficacy for cancer patients.^14,68^

## 9 Clinical Applications and Case Studies

Clinical Linking theoretical study with actual healthcare in real-world situations depends on clinical applications and case studies. These uses involve applying research results in standard medical procedures to improve patient outcomes and treatment success. Targeting nanoparticles and other innovations in drug delivery have altered our approach to administer medications. They decrease side effects and increase accuracy. Case studies provide vital new perspectives on how these innovative approaches apply in particular patient situations. They provide actual evidence of their advantages as well as any likely drawbacks. Examining how nanoparticle-based medications cure certain tumors could expose a wealth of information on changes in tumor size, patient responses, and survival rates generally. These investigations enable the identification of optimal approaches for patient treatment, improvement of operations, and resolution of arising issues in procedures. By applying actual data from real-life events, they also assist to develop medicine recipes and delivery systems. Combining case studies with therapeutic uses helps medical professionals better grasp and use innovative findings. It advances the overall area of medicine, stimulates fresh ideas in medical practices, and helps them provide better treatment for patients.^69^

### 9.1 Preclinical Studies and Findings

Preclinical research is critical for evaluating new drugs and technologies before they are tried in human trials. These investigations typically involve lab tests and animal models to determine the safety, efficacy, and mechanisms of action of novel medications. Preclinical research findings frequently contain information regarding pharmacokinetics, which deals with how a medication is absorbed, transported, metabolized, and excreted, as well as pharmacodynamics, which looks into the drug’s biological effects and causes. For example, preliminary research on a novel medicine may reveal that it can target specific cancer cells while inflicting minimal harm to normal tissue. This provides useful information about the potential therapeutic benefits and safety characteristics. These studies also help determine the appropriate dosing schedules and potential adverse effects, which are critical for future clinical trials. Furthermore, preclinical research can identify key biomarkers that can be used to assess the efficacy of a treatment or anticipate patient outcomes. Preclinical studies provide us with a fundamental grasp of how a treatment works and its effects in controlled environments. This guarantees that only the most promising and safe candidates go to human trials, improving the likelihood of favorable outcomes while decreasing patient risks.^69^

## 10 Clinical Trials and Human Studies

The development of medical knowledge and the manufacture of effective medications depend on the progress of which depends on clinical trials and human investigations. These carefully thought-out studies evaluate the safety, efficacy, and possible side effects of new drugs, treatments, or medical equipment. Usually following a methodical approach starting with preclinical research, they gather first data by means of laboratory and animal studies. After achieving successful completion, the study advances to clinical trials that involve human volunteers, which are categorized into multiple phases. Phase I trials primarily aim to evaluate the safety and dose of the intervention, typically involving a limited number of healthy volunteers.

Phase II trials aim to assess the effectiveness and potential adverse effects of a treatment in a wider cohort of individuals with the specific medical condition being targeted. Phase III trials encompass bigger cohorts to validate efficacy, monitor side events, and compare the novel treatment to established alternatives. Upon achieving success, Phase IV trials may be conducted subsequent to the marketing phase in order to collect extensive data regarding the intervention’s performance and safety within the broader population. Human research adheres to rigorous ethical norms and laws to guarantee the safety of participants and obtain their informed permission. They offer essential perspectives that stimulate medical advancement, directing clinical procedures and enhancing patient results.^31,70^

### 10.1 Overview of Ongoing and Completed Clinical Trials

Both ongoing and finished clinical trials are essential in the advancement of novel medical treatments and cures. Current trials are at different phases of advancement, ranging from initial-phase investigations examining safety and dose to final-phase trials reviewing effectiveness and long-term results. These trials frequently include many centers and various patient populations to guarantee thorough data collection and the capacity to apply the results to a wider population. Participants are closely observed by researchers to identify any negative effects, and if needed, the study protocols may be adjusted or the trial may be terminated early based on interim assessments. Conducted clinical trials make a substantial contribution to medical understanding by offering evidence regarding the efficacy and safety of therapies. The findings of these trials provide critical information for formulating clinical guidelines, regulatory approvals, and therapeutic practices. People frequently analyze and combine studies in systematic reviews and meta-analyses. This provides a better grasp of the overall benefit-risk profile of a medicine. In addition, completed studies can reveal areas that require additional research or previously undisclosed negative effects. Both ongoing and finished studies are critical to advancing medical science and improving patient care. They rely on solid procedures, ethical standards, and transparency to ensure that the results are reliable and appropriate for real-world scenarios.^31,70^

### 10.2 Evaluation of the Clinical Outcomes and Safety Profiles

Assessing the clinical outcomes and safety profiles is an essential aspect of both clinical trials and postmarketing surveillance. Clinical outcomes pertain to the efficacy of a treatment in attaining targeted health outcomes, such as amelioration of symptoms, remission of disease, or overall survival. These outcomes are assessed using different end points, which can include objective indicators like as laboratory findings or subjective reports from patients about their quality of life. An examination of these results aids in determining if a treatment offers a significant advantage over current alternatives or a placebo. Safety profiles, however, entail evaluating the negative effects and potential dangers linked to a particular medication. This is the surveillance of any severe, moderate, or small adverse reactions that could potentially affect the well-being or quality of life of the patient.

Safety data is obtained via regular clinical monitoring, patient reports, and follow-up examinations. Thorough safety profiles are crucial for comprehending the balance between the risks and benefits of a treatment and for directing its proper utilization. Continuous evaluation of both clinical outcomes and safety profiles is essential for obtaining regulatory approval and guiding clinical practice in clinical trials. Postmarketing studies enhance these evaluations by collecting real-world data, which aids in finding infrequent or long-lasting negative effects and assuring continuous patient safety.^31,70^

### 10.3 Case Studies of Successful Nanodrug Delivery in Cancer Immunotherapy

Successful case studies of nanodrug delivery in cancer immunotherapy demonstrate notable progress in precisely targeting and treating cancers. Nanodrug delivery systems employ nanoparticles to directly transport therapeutic drugs to cancer cells, yielding encouraging outcomes in enhancing treatment effectiveness and minimizing treatment adverse effects. These systems improve the ability of drugs to dissolve, remain stable, and be released in a controlled manner, resulting in the best possible therapeutic results. A such example is the use of liposomal nanoparticles containing chemotherapeutic medicines or immunomodulators. The nanoparticles are engineered to specifically target cancer cell markers, enabling for precise drug delivery to the tumor site while minimizing damage to healthy tissues. Clinical investigations have demonstrated that these formulations result in increased drug concentration in tumors, enhanced immune responses, and lower toxicity across the body.^31,70^

Using dendritic cell-based vaccines in conjunction with nanoparticles containing ant and adjuvants is another smart tactic. These systems effectively trigger the immune system to locate and destroy cancer cells, therefore reducing tumors and extending the lives of certain people. In essence, these case studies highlight how nanodrug delivery systems might alter cancer immunotherapy making targeting more exact, reducing side effects, and enhancing how well therapies work. They present a promising road forward for further approaches of cancer treatment.

### 10.4 Detailed Analysis of Case Studies Where Nano-DDS Were Used Successfully

An in-depth examination of case studies demonstrating the successful use of nanodrug delivery systems (-DDS) reveals some crucial findings about their efficacy and potential. One notable case study investigated lipid-based nanoparticles for the targeting of doxorubicin, a widely used chemotherapeutic medication, precisely to breast cancer cells. These nanoparticles were designed to improve the drug’s capacity to dissolve and remain stable while also targeting certain indicators on the cell surface. The clinical results revealed a significant increase in medication concentration within the tumor. This results in higher treatment efficacy and fewer harm to the body than normal doxorubicin therapy. Another major success was the use of polymeric nanoparticles containing checkpoint inhibitors to treat cancer. The nanoparticles were designed to gently release checkpoint inhibitors, thereby boosting the immune response to cancer cells. Clinical trials revealed higher tumor response rates (and fewer side effects), which were typically associated with systemic distribution of these inhibitors.^31,70^

## 11 Challenges and Future Directions

First and foremost, delivering nanoparticles to the proper tumor cells is a difficult task. Targeted nanotherapy’s efficacy is typically limited by tumor heterogeneity and numerous cellular and molecular obstacles. To overcome these obstacles, you must have a thorough understanding of tumor biology. You also need to create smart nanoparticle architectures that can navigate the tumor microenvironment. Achieving this level of accuracy remains a major issue. Furthermore, it is difficult to prevent nanoparticles from being absorbed by nontarget organs. This could result in unforeseen consequences and toxicity.^71^

## 12 Regulatory and Ethical Considerations

The inclusion of nanotechnology into cancer immunotherapy has enormous promise for revolution. However, regulatory and ethical considerations must be taken into account. Understanding and adhering to a complicated set of regulations and criteria is required for regulating nanotechnology in drug delivery systems. The United States Food and Drug Administration (FDA) and the European Medicines Agency (EMA) have developed methods to analyze nanomedicines, taking into account their particular characteristics and special challenges. The FDA’s Nanotechnology Task Force has issued guidance on analyzing nanomaterials, with an emphasis on their physicochemical qualities, potential toxicity, and biological interactions. The issue is to adapt present regulatory frameworks to the evolving properties of nanoparticles and their various effects on human health and the environment. Furthermore, consistent testing methodologies are required to ensure the safety and efficacy of nanotechnology-based medication delivery systems. Existing protocols may not sufficiently account for the complexities of these complicated medicines.^72,73^

## 13 Future Perspectives

**Figure 18:**
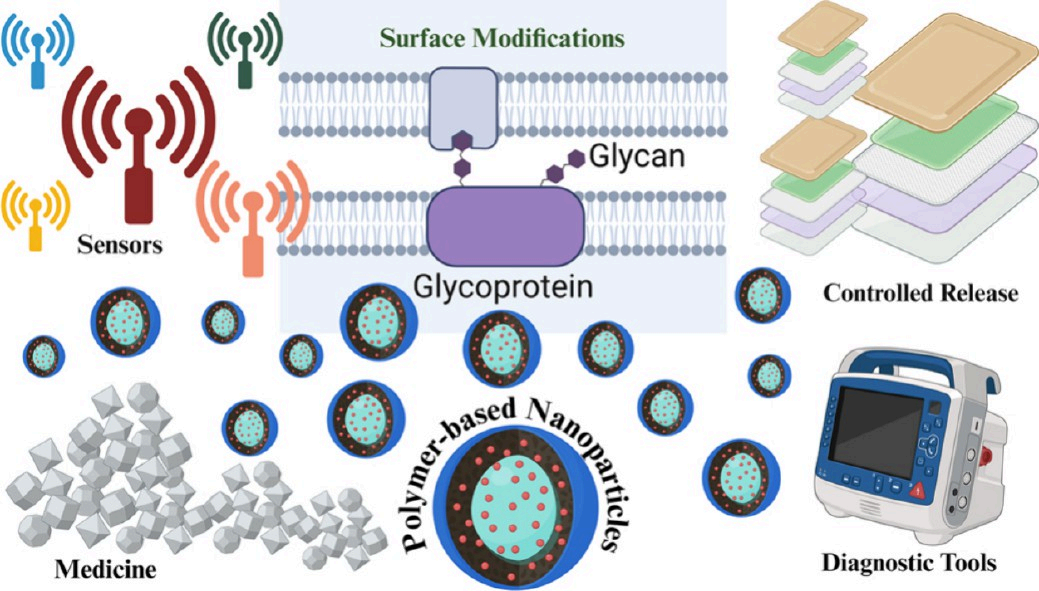
Figure illustrates various applications of polymer-based nanoparticles, including their roles in sensors, surface modifications, controlled release mechanisms, diagnostic tools, medicine and potential biomedical uses.

New nanotechnology-driven medication delivery techniques have great potential to produce major future innovations in cancer immunotherapy. It is believed that as nanotechnology develops it will surpass many of the main constraints of present cancer treatments, hence enhancing their efficacy and targeting power. Advancement of multifunctional nanoparticles is one quite interesting field of study Figure 18. Like immunomodulatory drugs, chemotherapeutic medications, and targeted antibodies, these nanoparticles can transport several therapeutic agents concurrently. One can control them such that their contents are methodically and under control released. This method reduces any accidental negative effects and maximizes the intended therapeutic results.^74^

## 14 Discussion

Here, the discussion of this review focuses on a powerful way to extend such frontiers—de novo bioconjugation for conjugative theranostic cancer immunotherapy and dispenses with nanotechnology-driven drug delivery systems (DDS). Even though immunotherapy—immune checkpoint inhibitors and CAR T-cell therapy—provides minimal survival outcomes, its efficacy in treating certain patient groups is limited and it is associated with toxicity. Nanotechnology helps to improve delivery of drugs with more precision and efficacy where it provides targeted treatment which reduces harm to other normal tissues. This can be further enhanced by advances in nanoparticle construction (lipid-based, polymeric, or inorganic) that not only provide drug stability but allow for the codeliver of therapeutic agents leading to better treatment outcomes. The review was focused on one point: the safety and efficacy of Nano-DDS in preclinical and clinical trials, indicating that it may improve the therapeutic index of anticancer drugs. The progress is accompanied by recognition of barriers that need to be overcome to realize the full potential of nanotechnology, such as regulatory and methodological hurdles. Research in these areas of barriers need to be broken if we are going to move forward with personalized and nanotechnology-enabled cancer therapies.

## 15 Conclusion

The inclusion of nanotechnology into cancer immunotherapy represents a significant shift in cancer treatment. Nanotechnology-based drug delivery has the potential to change medicine by addressing the limitations of traditional treatments. These systems seek to increase therapy efficacy while minimizing side effects. They are distinguished by their unrivalled capacity to administer medications with pinpoint accuracy, targeting cancers while sparing healthy tissue.

Nanotechnology in cancer immunotherapy greatly improves drug delivery precision and control. Nanoparticles can be designed to contain a variety of medicinal chemicals, including chemotherapeutic medicines, immunomodulators, and particular antibodies. These compounds can then be released in a regulated fashion directly at the tumor location. This targeted administration not only improves the treatment’s therapeutic impact but also lowers overall toxicity, addressing a major drawback of standard therapy. Furthermore, the capacity to personalize nanomedicines based on specific tumor characteristics represents a substantial advancement in cancer treatment. Higher therapeutic precision and efficacy can be achieved by customizing nanoparticles to the particular characteristics of a patient’s cancer cells. This tailored strategy could alter cancer treatment by increasing the likelihood of effective outcomes and reducing the trial-and-error associated with standard drugs.

Nanotechnology shows promise in tackling tumor microenvironmental issues. Advances in nanoparticle design are improving their ability to enter and aggregate in tumors, overcoming obstacles such as high interstitial pressure and poor vascularization. This improved delivery can lead to more effective therapy and greater therapeutic outcomes. Furthermore, combining real-time imaging and monitoring technology with nanomedicine is expected to alter how cancer medicines are delivered. By giving information about nanoparticle distribution and behavior throughout the body, medical professionals can optimize treatment regimens and modify medicine doses in real time, resulting in more precise and successful therapy.

The development of safer and more biocompatible nanoparticle materials increases the promise of nanotechnology-based medication delivery systems. Developing biodegradable and bioresorbable materials is intended to reduce side effects and improve patient safety, ultimately boosting treatment acceptance and effectiveness. However, putting these advances into clinical practice necessitates extensive testing and validation. Effective collaboration among researchers, physicians, and industry stakeholders is critical for extending new technologies while guaranteeing their safety and efficacy across diverse patient populations.

Finally, nanotechnology has made significant advances in cancer immunotherapy. Ongoing research and new technologies make it increasingly possible that nanotechnology-based drug delivery systems will have a substantial impact on cancer treatment. These technologies could provide more efficient, personalized cancer therapies, indicating significant progress against this difficult condition. Further research and advancement of these technologies are projected to usher in a new era of cancer treatment, characterized by improved patient outcomes and renewed hope for individuals impacted by cancer.

The significance of nanomaterials to combine with cancer immunotherapy, in order to improve current obstacles and clinical outcomes. Immunotherapy, including immune checkpoint inhibitors and CAR T-cell therapy, has demonstrated strong efficacy in cancers such as melanoma and non-small-cell lung cancer; however, these modalities face several challenges, for instance differing response rates among patients and toxicities associated with beneficial effects. Nanotechnology based solutions, allowed for targeted and controlled drug delivery, enhanced drug stability in vivo resulting in improved oral bioavailability and reduced systemic dose side-effects. The authors of the review highlight the potential of delivery systems in nanotechnology (with lipid, polymeric, or inorganic nanoparticles), namely alone and/or by combination with immunotherapy to increase effectiveness in oncology. However, the review also highlights the difficulties and regulations that nanotechnology may encounter during clinical practice, and it highlights further studies required to improve personalized therapeutics in case of cancer. Nanotechnology as a whole will be able to substantially improve the therapeutic index of cancer treatments and overcome current obstacles in cancer immunotherapy.
